# The Validation and Accuracy of Wearable Heart Rate Trackers in Children With Heart Disease: Prospective Cohort Study

**DOI:** 10.2196/70835

**Published:** 2025-09-30

**Authors:** Hidde J Hardon, Yara N Van Kerkhof, Beatrijs Bartelds, Janneke A E Kammeraad, Arend W Van Deutekom

**Affiliations:** 1 Division of Pediatric Cardiology, Department of Pediatrics, Erasmus MC, Dr Molewaterplein 40, Rotterdam, 3015GD, The Netherlands, 31 10 704 0704

**Keywords:** wearable technology, heart rate monitoring, ECG, patient satisfaction, electrocardiogram, validation

## Abstract

**Background:**

Wearables are increasingly used in pediatric cardiology for heart rate (HR) monitoring due to advantages over traditional HR monitoring, such as prolonged monitoring time, increased patient comfort, and ease of use. However, their validation in this population is limited.

**Objective:**

The objective of this paper was to assess HR accuracy and validity from 2 wearables, the Corsano CardioWatch bracelet and the Hexoskin smart shirt, in children attending the pediatric cardiology outpatient clinic, exploring factors that influence accuracy, the Hexoskin shirt’s arrhythmia detection efficacy, and patient satisfaction.

**Methods:**

Children with an indication for 24-hour Holter monitoring were equipped with a 24-hour Holter electrocardiogram (ECG; gold standard), together with both wearables. HR accuracy was defined as the percentage of HRs within 10% of Holter values, and agreement was assessed using Bland-Altman analysis. Subgroup analyses were conducted based on BMI, age, and time of wearing, among other factors. The association between accelerometry (expressed in gravitational units, g) and HR accuracy was analyzed to assess the impact of bodily movement on measurement accuracy. A blinded pediatric cardiologist analyzed Hexoskin shirt data for rhythm classification. Patient satisfaction was measured using a 5-point Likert scale questionnaire.

**Results:**

A total of 31 participants (mean age 13.2, SD 3.6 y; n=14, 45% female) and 36 participants (mean age 13.3, SD 3.9 y) were included for the CardioWatch and Hexoskin measurements, respectively. Mean accuracy was 84.8% (SD 8.7%) for the CardioWatch and 87.4% (SD 11%) for the Hexoskin shirt. Hexoskin shirt accuracy was notably higher in the first 12 hours (94.9%, SD 7.4%) compared to the latter 12 (80%, SD 16.7%; *P*<.001). Higher accuracy was observed at lower HRs (low vs high HR: CardioWatch: 90.9%, SD 9.3% vs 79%, SD 10.6%; *P*<.001 and Hexoskin shirt: 90.6%, SD 14% vs 84.5, SD 11.8%; *P*<.001). Both wearables demonstrated good agreement in their HR measurement with Holter readings (CardioWatch bias: –1.4 beats per minute [BPM]; 95% limits of agreement [LoA] –18.8 to 16.0. Hexoskin shirt bias: –1.1 BPM; 95% LoA −19.5 to 17.4). HR measurement accuracy declined during more intense bodily movements. Correct classification of the Hexoskin’s shirt rhythm recordings was achieved in 86% (31/36) of cases. Patient satisfaction scores were significantly higher for both the CardioWatch (median 3.8, range 3.5‐4.3; *P*<.001) and Hexoskin shirt (median 3.7, range 3.0‐4.0; *P*<.001) compared to the Holter (median 2.6, range 2.1‐3.2).

**Conclusions:**

The Corsano CardioWatch and Hexoskin shirt demonstrate good accuracy in pediatric HR monitoring and provide higher patient comfort than conventional monitoring. Both wearables show good agreement in relation to the gold standard device. However, measurement accuracy declines with increasing bodily movement and higher heart rates. More research is needed to explore the underlying causes for these inaccuracies and how to counteract them. The Hexoskin shirt also shows potential in arrhythmia detection. While further development is warranted, these wearables show promise in enhancing diagnostics, therapeutic monitoring, and patient safety in pediatric cardiology.

## Introduction

The Holter electrocardiogram (ECG; Spacelabs Healthcare) is widely recognized as the gold standard for ambulatory monitoring of heart rate and rhythm in children [1,2]. However, its bulkiness, the potential for skin irritation caused by plaster electrodes during prolonged use, and the loose wires make it a cumbersome diagnostic tool for extended monitoring, particularly in children. In addition, infrequently appearing symptoms may result in a low diagnostic yield [3,4] and often necessitate extended or repeated monitoring [5]. The multiple hospital visits that are often needed for cardiac monitoring further affect the child’s quality of life [6]. This highlights the need for innovative technologies that can monitor heart rate in children reliably over extended periods of time, are comfortable to wear, and that can preferably be used at home. Ideally, these tools should also allow for remote data access, which could enable timely medical interventions when disease deterioration is detected or reassurance for the patient and family.

Recently, wearable devices, defined as biosensors that can be worn on the body to monitor various health parameters [7], have become increasingly popular and show potential for the follow-up care of patients with heart conditions [8]. Some devices have already been explored in cardiovascular care, particularly for heart rhythm diagnostics [9], such as atrial fibrillation [10], and may offer significant benefits in medical settings, especially for patients with (congenital) heart disease [11,12]. However, most data on wearable validity comes from studies including only adult patients [13,14]. This validity cannot be extrapolated to children, as children have distinct physiological and behavioral properties. For example, differences in wearable fit, children’s high-intensity activity patterns, and higher, more variable heart rates—often exceeding 200 beats per minute (BPM)—may impact measurement quality and accuracy [15,16]. This should all be considered when aiming to use wearables in the pediatric setting and highlights the need for more validated research that includes the younger population.

Therefore, this study investigates the validity of 2 recently developed wearable devices in children: a wristband that measures heart rate and an ECG shirt that records heart rhythm and heart rate. Validation is performed in a cohort of children with congenital heart disease (CHD) or (suspected) arrhythmias, during a 24-hour free-living period, in comparison with the gold standard Holter ECG. In addition, this study aims to explore factors that influence measurement accuracy, such as bodily movement, and evaluates patient comfort and satisfaction with the wearables during the 24-hour measuring period.

## Methods

### Recruitment

Patients between 6 and 18 years of age, attending the outpatient clinic from the pediatric cardiology department of Erasmus MC Sophia Children’s Hospital, with an indication for 24-hour or 48-hour Holter ECG monitoring were invited to participate in this study. Before the scheduled Holter monitoring, the participant’s health care provider contacted the patient’s family to inform them about the research. An information letter was sent out via email. During their hospital visit, the child and family were asked to participate. In case of a positive answer, informed consent forms (see the Ethical Considerations section) were signed, and the patients were outfitted with the wearables. Children younger than 6 years of age were excluded because, at the time of inclusion, wearable sizes for younger children were not available. Another exclusion criterion was the inability to cooperate.

### Ethical Considerations

The study was approved by the Institutional Review Board of the Erasmus MC (MEC-2022‐0576 and MEC-2022‐0499) and all participants aged 12 years and older and the legal guardians gave informed consent before participation. Children aged 12 years and younger provided assent. The informed consent forms were adapted to the level of understanding of the children. Data were pseudo-anonymized with the key table only accessible for members of the research team. Participants did not receive financial compensation, as the study procedures were integrated into routine clinical care and imposed only minimal additional burden.

### Research Devices

The Holter ECG device (Spacelabs Healthcare), hereafter “Holter,” is a medical-grade 3-lead ECG device known as the gold standard in ambulatory heart rate and rhythm measuring [2] and was therefore used as a criterion measure to investigate the validity of the heart rate measured by the Corsano CardioWatch 287-2B, hereafter “CardioWatch,” and the validity of the heart rate and heart rhythm registered by the Hexoskin Pro shirt (Carré Technologies Inc), hereafter “Hexoskin shirt.” The CardioWatch is a CE medically certified multisensor wristband for continuous, in-hospital, and remote patient monitoring to detect abnormal heart rates [17]. The CardioWatch uses reflective photoplethysmogram (PPG) signals to measure heart rate, R-R intervals, and respiration rate.

The Hexoskin shirt is a commercially available smart garment that measures ECG signals using 3 sensors that are woven into the fabric [18]. The sensors, with 2 positioned on either side at the level of the serratus anterior muscles and 1 on the right side of the abdominal muscles, register a single-lead ECG, capturing heart rhythm, heart rate, and heart rate variability.

In addition, both wearables are equipped with a built-in accelerometer that measures bodily movements.

### Procedure

Both wearables were charged before the measurement and were set up with a new patient study account. After obtaining informed consent, the participant’s demographic and anthropometric characteristics and their clinical indication for Holter monitoring were recorded, and both wearables and Holter were placed on the participant’s body. The Holter electrodes were placed by a certified nurse, following the usual protocol. To prevent overlap between the Holter electrodes and the electrodes integrated in the Hexoskin shirt, the Holter electrodes, which could block the measurement of the Hexoskin shirt, were placed slightly more toward the sternum. Next, the participants were outfitted with both the CardioWatch wristband and Hexoskin shirt. First, the CardioWatch was fitted tightly, but comfortably around the nondominant wrist and was subsequently connected via Bluetooth to the researcher’s smartphone. Once connected, a new recording session was started in the manufacturer’s proprietary smartphone app. The smartphone was given to the participant or legal guardians with the instruction to keep it close, within 10 meters, to the CardioWatch for the 24-hour measuring period to allow for good data synchronization. Finally, the Hexoskin shirt was put on, with the appropriate shirt size based on the participant’s chest circumference. After visually checking for interference between the Holter and the Hexoskin shirt’s electrodes, transmission gel was applied to the Hexoskin shirt’s electrodes to allow for better signal conduction, as recommended by the manufacturer. The Hexoskin shirt was then connected with the Hexoskin pro device, which initialized recording of data. The data were visually checked in the manufacturer’s smartphone app on the researcher’s smartphone. No further calibration steps were needed before measuring. Participants were encouraged to live their normal daily routine but refrain from showering and swimming.

In line with the standard protocol for the 24- and 48-hour Holter measurements, the child was asked to fill in a diary with information about activities, the appearance of any symptoms, and bedtimes. All devices were removed by the patient or legal guardians 24 hours after the start of the measurement and returned to the hospital together with the smartphone and diary. For participants undergoing 48-hour Holter monitoring, only the wearable devices were removed after 24 hours, while the Holter measurement continued for the full 48-hour period. To investigate the perceived comfort of both wearables and the Holter device, patients filled in a Likert-scale questionnaire with questions related to comfort, ease of use, and impact on sleep (self-developed, see Multimedia Appendix 1 for the questionnaire [Dutch] or Multimedia Appendix 2 for the overview of questions [English]).

### Data Acquisition

After synchronization, data obtained by the CardioWatch (firmware version 4.51) were automatically uploaded to the manufacturer’s web application (Corsano Portal; version 1.0.3) via the accompanied smartphone. The manufacturer’s proprietary algorithm calculates heart rate in BPM from raw PPG signals. These data in BPM were then downloaded from the online portal for further analysis.

In a similar way, BPM data from the Hexoskin shirt were downloaded from the Hexoskin web dashboard after synchronization of the pro device—which stored the measurement data—with the manufacturer’s browser-based app (version 5.0.12.348; OneSync).

To investigate the association between bodily movement and measurement accuracy, raw accelerometry data of both wearables were downloaded. For the CardioWatch, the raw triaxial accelerometry data (acc_x, acc_y, and acc_z) is captured in unit 1/512 g. The intensity of the movement in g’s was derived from the raw accelerometry data divided by 512, and the following equation [19]:


$$\begin{array}{l} d\_accX=acc\_x[i]-acc\_x[i-1];d\_accY=acc\_y[i]-acc\_y[i-1];\\ d_{a}ccZ=acc\_z[i]-acc\_z[i-1];\\ Norm\_of\_d\_acc=sqrt(d_{a}ccX*d_{a}ccX+d_{a}ccY*d_{a}ccY+d_{a}ccZ*d_{a}ccZ) \end{array}$$

Here, *d_accX, d_accY, and d_accZ* represent the change in acceleration along the X, Y, and Z axes between two consecutive time points, while *acc_x[i], acc_y[i], and acc_z[i]* represent the raw acceleration values at time point *i* along each respective axis. *Norm_of_d_acc* reflects the magnitude of the change in acceleration across all directions.

The resulting accelerometry intensity in g was used for further analysis. For Hexoskin, the accelerometry data were already calculated in unit g, as recorded by the Hexoskin pro-device.

Holter data were analyzed, following standard procedure, by a Holter specialist and were subsequently downloaded from the Erasmus MC database using Sentinel software (version 11.5.5.14400; Spacelabs Healthcare). Heart rate was derived from the R-R intervals and calculated as a moving average over the previous 16 beats to mitigate the impact of artifacts and noise. To adjust for differences in the devices’ time settings, the optimal offset was calculated as the highest cross-correlation between the Holter and wearable device within 60 seconds and adjusted using a Python script (Python Software Foundation). The CardioWatch and Hexoskin shirt data were interpolated to the criterium measurement data and filtered for further analysis. BPM data of all three devices were used for analysis.

### Missing Data

Missing heart rate data of the CardioWatch and Hexoskin shirt were considered underdetected, missing heart rate data of the Holter were considered erroneous due to malfunctioning of the criterion measure. To allow for accurate comparison of data, erroneous data were excluded from the analysis.

### ECG Rhythm Registration

Holter ECG registrations were analyzed by a Holter specialist, using software to detect clinically relevant parts of the recording. These mostly include normal sinus rhythm (SR), supraventricular tachycardia (SVT), ventricular tachycardia (VT), bradycardia, and single extra beats, such as premature atrial contractions (PAC) or premature ventricular contractions (PVC), among others. To see if the Hexoskin shirt could detect these arrhythmias, its ECG rhythm recordings were presented to a pediatric cardiologist. The rhythm recordings made by the Hexoskin shirt were accessed online through the Hexoskin dashboard. After analysis of the Holter device by the Holter specialist, clinically relevant ECG rhythms were selected and compared to the readings from the Hexoskin shirt. In total, 36 of these signals were then presented to a blinded pediatric cardiologist for interpretation. For each Hexoskin recording, the corresponding Holter rhythm from the same timeframe was included, resulting in a total of 72 rhythms for review—36 from the Hexoskin shirt and 36 from the Holter monitor. The Holter data were presented without previous interpretation from the Holter specialist, ensuring unbiased classification. Hexoskin and Holter recordings were presented in a random order.

### Statistical Analysis

#### Overview

Descriptives are shown as mean (SD) or as median IQR when appropriate. Differences between subgroups were analyzed using independent sample *t* tests or ANOVA tests in the case of more than 2 groups. Within-group effects were analyzed using paired sample *t* tests. Normality of distributions of data was checked both visually and with the Shapiro-Wilk test. In case of any violation of normality, the data were analyzed using nonparametric equivalent tests. The significance level was set at *P*<.05 (2-tailed). All statistical analyses were performed using SPSS (version 28.0.1.0(142); IBM Corp) and Python (version 3.10.9).

#### Accuracy

Measurements within 10% of criterion measurement values were defined as accurate, which is in line with the Association for the Advancement of Medical Instrumentations (AAMI) guidelines [20].

#### Mean Absolute Error and Mean Absolute Percentage Error

Mean absolute error (MAE) was calculated as the MAE between wearable and criterion measure heart rate measurements. Mean absolute percentage error (MAPE) was calculated as the mean absolute error between wearable and criterion measure heart rate data, divided by the criterion measure’s value and multiplied by 100. Individual MAPEs were averaged to obtain an overall MAPE for each wearable. The absolute error between the criterion measure and the wearable measure is used to calculate MAPE, as this approach accounts for both overestimation and underestimation of the measurements. The equations to calculate these measures of error are shown in Multimedia Appendix 3. As described by Nelson et al [21], a MAPE score of <10% was defined as accurate.

#### Bland-Altman Analysis

The Bland-Altman analysis was used to analyze the agreement between wearable devices and Holter measurements. The mean differences are reported with 95% limits of agreements (LoA). Bland-Altman plots were created to visualize the agreement.

#### Lin Concordance Correlation Coefficient (CCC)

Correlation between both wearables and Holter was assessed separately using Lin concordance correlation coefficient (CCC) and averaged over all participants to form a total CCC for each wearable [22]. Agreement strength was scored as weak (CCC<0.5), moderate (CCC=0.5‐0.7), and strong (CCC>0.7), as in line with previous research [23].

#### Patient Satisfaction Scores

Patient satisfaction was measured using a 5-point Likert scale questionnaire, which was developed for the purpose of this study (see Multimedia Appendix 1 [Dutch] or Multimedia Appendix 2 [English]). The questionnaire outcomes were analyzed using nonparametric Wilcoxon tests. Wear time was included as a quantitative usability metric.

#### Accelerometry Analysis

To investigate the association between bodily movement and the accuracy of HR measurements, the accelerometry data of both the CardioWatch and the Hexoskin shirt were analyzed. As there is no clear consensus on physical activity intensity thresholds (in gravitational units) in children, and both wearables measure different bodily movements (ie, wrist-based for the CardioWatch and torso-based for the Hexoskin), the accelerometry analysis was based on individual thresholds of both devices. Thereby, the HR measurement accuracy was calculated based on the quantiles of the participants’ own accelerometry data, where lower quantiles correspond to fewer bodily movements. Mean accuracy scores per quantile were computed across all participants to create an overall accuracy score per quantile for each wearable.

### Subgroup Analysis

Based on our pilot study, which showed a relationship between wrist circumference and accuracy of the CardioWatch, wrist circumference was included as a covariable. Next to that, the effect of sex, age, BMI, skin type, time of day, heart rate frequency, and measuring period on the accuracy of heart rate measurements was investigated. Subgroups were created with the following thresholds: age (≤12 and >12 y), BMI (≤19 and >19 kg/m^2^), wrist circumference (≤15.5 and >15.5cm, for CardioWatch only), time of day (wake time and sleep time), heart rate (above or below median heart rate), and measuring period (first 12 h and second 12 h of measuring). The subject’s median heart rate was chosen as the threshold between lower and higher heart rates instead of the mean heart rate, as the mean heart rate is influenced by outliers. Skin type differentiation (Fitzpatrick I and II, Fitzpatrick III, and Fitzpatrick IV, V, and VI, for CardioWatch only) was included as darker skin is known to influence the optical PPG measurement [24,25]. To see if the patient’s diagnosis had an influence on heart rate and rhythm accuracy, participants were grouped according to the indication for their Holter measurement: CHD, known arrhythmia, or symptoms (eg, palpitations or fainting). To accurately delineate the transition between wakefulness and sleep, the 60 minutes around reported sleep times were not included in sleep vs wake accuracy analysis. The diary information was used to differentiate between wake and sleep times.

## Results

### Baseline Characteristics

A total of 39 participants took part in this study. Due to the malfunctioning of the criterion measure, data from 2 participants were excluded before analysis. For the CardioWatch, data of 6 participants were excluded from analysis due to either early loss of battery power (n=1), an accidental reset of the CardioWatch during the measurement (n=1), or an accidental termination of the smartphone app (n=4), which all resulted in the loss of data. The data of the remaining 31 participants were included for analysis. For the Hexoskin shirt, the data of 1 participant was excluded because the shirt was taken off after 2 hours of measuring. The data of the remaining 36 participants were included for analysis. In total, 38 out of the 39 participants who completed the 24-hour measurement filled in the questionnaire. All were included in the questionnaire analysis. Table 1 shows the participants’ characteristics.

**Table 1.** Participant characteristics. Wrist circumference and Fitzpatrick outcomes were not used for the Hexoskin shirt analysis.

Participants characteristics	CardioWatch (n=31)	Hexoskin shirt (n=36)
Characteristics		
Sex: female, n (%)	14 (45)	16 (44)
Age (years), mean (SD)	13.2 (3.6)	13.3 (3.9)
Height (m), mean (SD)	1.60 (0.18)	1.59 (16.5)
Weight (kg), mean (SD)	52.2 (16.8)	50.8 (16.0)
BMI (kg/m^2^), mean (SD)	19.9 (3.7)	19.5 (3.6)
Wrist circumference (cm), mean (SD)	15.4 (1.7)	—^a^
Indication for Holter, n (%)		
Congenital heart disease	11 (36)	13 (36)
Arrhythmia	14 (45)	16 (44)
Symptoms	6 (19)	7 (19)
Fitzpatrick score, n (%)		
Type I	0 (0)	—
Type II	10 (34)	—
Type III	13 (45)	—
Type IV	4 (14)	—
Type V	2 (7)	—
Type VI	0 (0)	—

a Not applicable.

### Missing Data

Underdetected data or missing data of the CardioWatch were calculated as the total missing timepoints and occurrences of zeros in the data. Due to a bug in the CardioWatch software, data of the last 9 participants showed a shift in sample rate at night from once per second to one measurement per minute. This resulted in missing time points and therefore a high percentage of missing data (36%‐40%). These participants were included in accuracy measurements but excluded from the calculation of missing data and the accelerometry analysis. Mean percentage of underdetected data for the CardioWatch was 2.5% (SD 2.1%), ranging from 0.02% to 10.6%. Erroneous data, which was defined as missing datapoints in Holter data, was significantly lower with a mean of 1.3% (2.2%), ranging from 0.01% to 10.2%. The percentage of missing data for all participants is shown in Multimedia Appendix 4. The missing data percentage of the Hexoskin shirt could not be determined because a heartbeat of 70, or the last known heartbeat, is shown for missing datapoints.

### Accuracy of Heart Rate

For the CardioWatch, the overall accuracy of the 24-hour heart rate measurement ranged between 67.1% and 98.7% with a mean of 84.8% (SD 8.7%). For the Hexoskin shirt, the accuracy of the heart rate measurements was captured between 60.3% and 99.4% with a mean of 87.4% (SD 11%). Both wearables, therefore, demonstrate good accuracy in heart rate measurements (see Table 2), although with high interindividual variability (see Multimedia Appendix 5 for the individual accuracy scores per participant).

**Table 2.** Main study parameter outcomes for both the CardioWatch and the Hexoskin.

Parameters	CardioWatch (n=31)	Hexoskin (n=36)
Accuracy (%), mean (SD)	84.8 (8.7)	87.4 (11)
MAE^a^ (BPM^b^), mean (SD)	4.9 (1.8)	4.3 (3.2)
MAPE^c^ (%), mean (SD)	5.5 (2.2)	4.9 (3.8)
Agreement (BPM), median (IQR)	–1.4 (–18.8 to 16.0)	–1.1 (–19.5 to 17.4)
CCC^d^ (95% CI)	0.872 (0.869‐0.874)	0.845 (0.842‐0.848)
Comfort score (n=38), median (IQR)	3.8 (3.5‐4.3)	3.7 (3.0‐4.0)

a MAE: mean absolute error.

b BPM: beats per minute.

c MAPE: mean absolute percentage error.

d CCC: Lin concordance correlation coefficient.

### Error

The CardioWatch had an overall MAE and MAPE of 4.9 (SD1.8) BPM and 5.5% (SD 2.2%), respectively. The Hexoskin shirt showed an overall MAE and MAPE of 4.3 (SD 3.2) BPM and 4.9% (SD 3.8%). These results indicate that both wearables had a comparable average absolute error relative to the criterion measure. Similarly, both wearables showed a MAPE of around 5%, meaning that the average difference between measurements from the wearables and the Holter was around 5% of Holter measurement values. This error margin is well below the 10% threshold described by Nelson and colleagues [21] and indicates a small error and accurate measurement performance. An overview of all the individual scores is presented in Multimedia Appendix 6.

### Agreement

**Figure 1.**
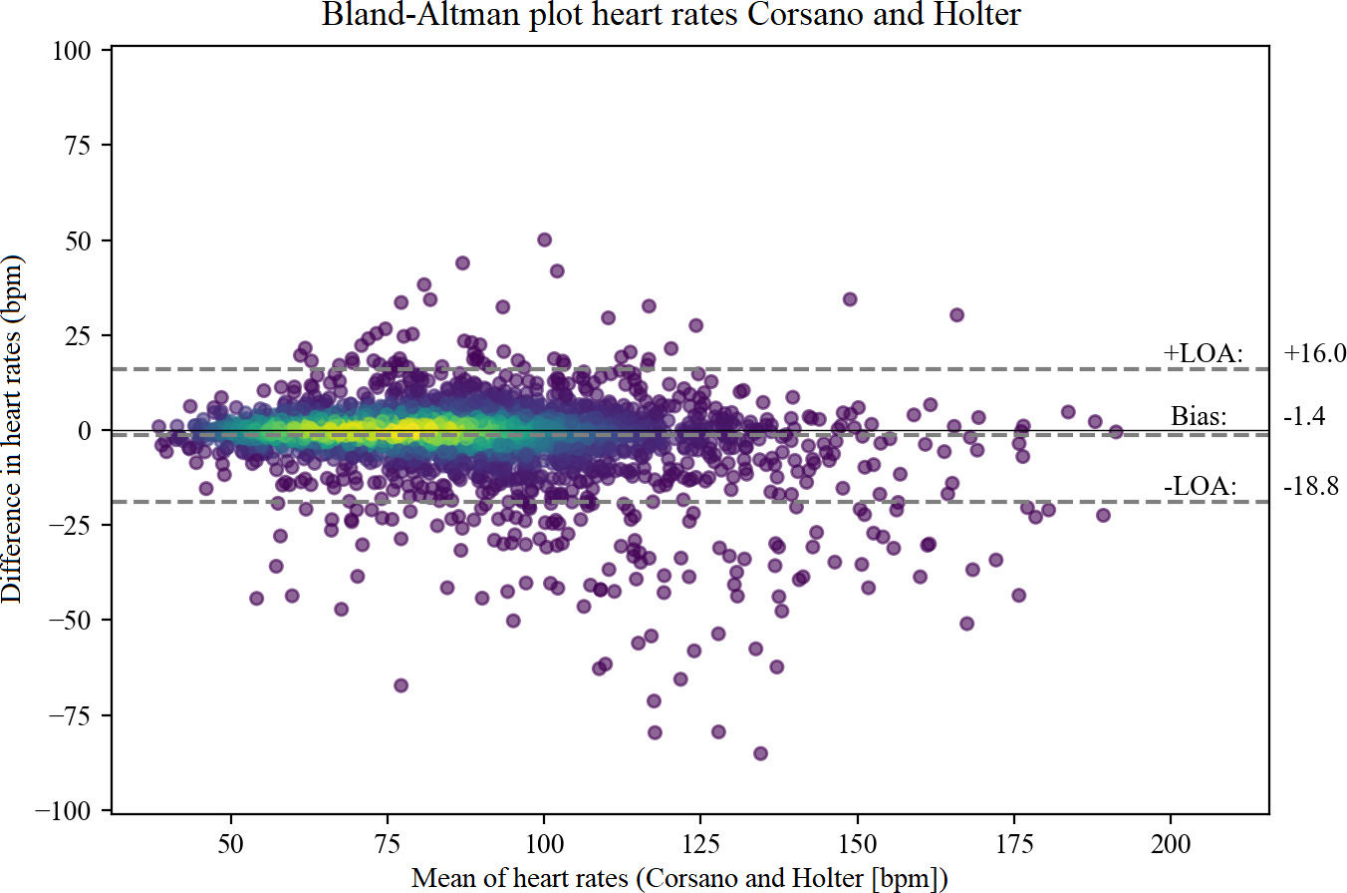
Bland-Altman plot CardioWatch. bpm: beats per minute; LOA: limits of agreement.

**Figure 2.**
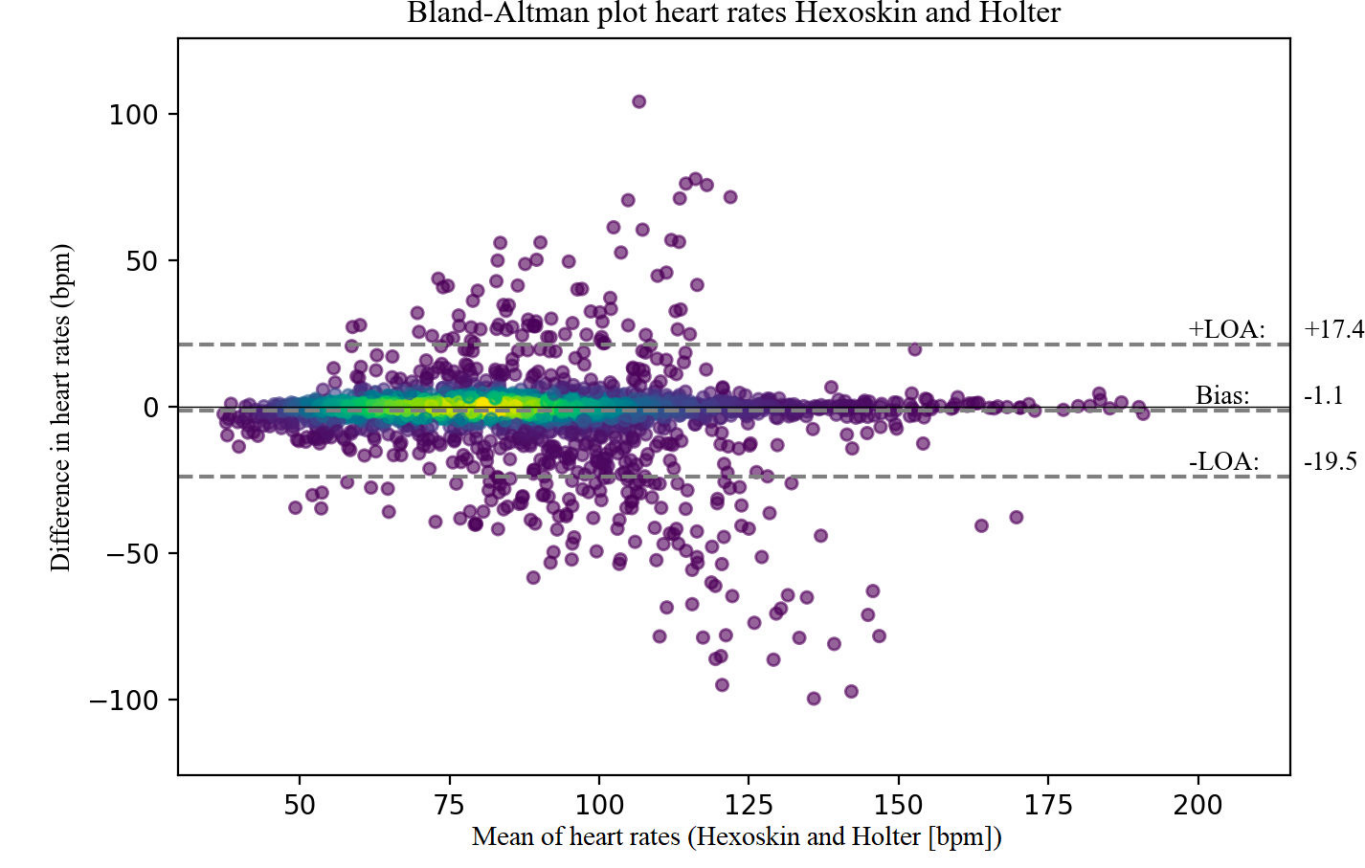
Bland-Altman plot Hexoskin. bpm: beats per minute; LOA: limits of agreement.

The CardioWatch had a slight underestimation of the HR measurements compared to the Holter, with a mean difference of −1.4 BPM, and 95% LoA ranging from −18.8 to 16.0 (see Figure 1). For the Hexoskin shirt, the Bland-Altman analysis showed a small underestimation of −1.1 BPM and 95% LoA ranging from −19.5 to 17.4 (see Figure 2).

### Concordance Correlation Coefficient

The CardioWatch had a CCC of 0.872 (95% CI 0.869‐0.874) and the Hexoskin shirt showed a CCC of 0.845 (95% 0.842‐0.848), both indicating strong agreement in relation to the criterion measure. Individual scores are shown in Multimedia Appendix 7.

### Accuracy of Heart Rhythm

**Figure 3.**
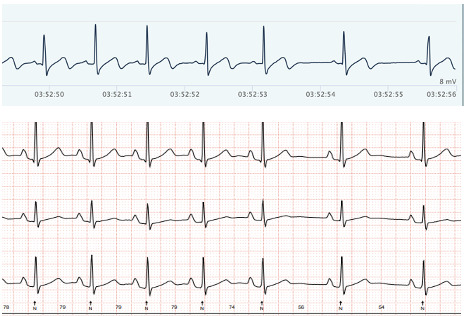
Example of a clear electrocardiogram (ECG) rhythm. Hexoskin recordings are depicted above and Holter ECG rhythms below.

Figures3^6^ show examples of the ECG rhythms recorded by the Holter monitor and the Hexoskin shirt. No significant rhythm abnormalities, such as SVTs or VTs, were detected during the 24-hour monitoring period for any of the participants. Minor abnormalities, including isolated extra beats, did occur. All 36 Holter recordings were accurately classified by the pediatric cardiologist. The Hexoskin shirt’s ECG recordings were correctly classified in 31 out of 36 cases. The remaining 5 recordings could not be classified with certainty due to artifacts and noise in the Hexoskin shirt’s ECG signal. The 2 examples of clear registrations are shown in Figures3  4. In addition, 2 examples of rhythms with noise, which impeded accurate classification, are shown in Figures5  6. None of the recordings were incorrectly classified as normal or abnormal. Multimedia Appendix 8 shows an overview of all the arrhythmias that were included in the rhythm recordings that were presented to the pediatric cardiologist.

**Figure 4.**
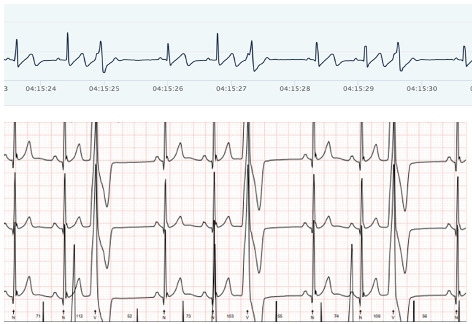
Example of a clear electrocardiogram (ECG) rhythm. Hexoskin recordings are depicted above and Holter ECG rhythms below.

**Figure 5.**
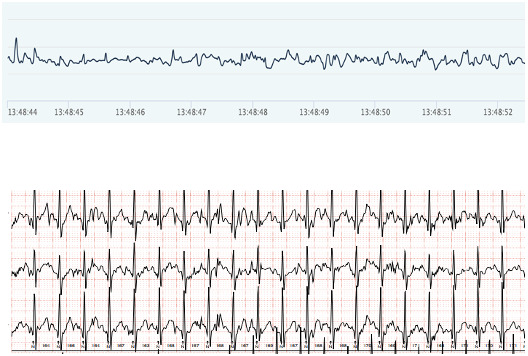
Example of electrocardiogram (ECG) rhythm with noise. Hexoskin recordings are depicted above and Holter ECG rhythms below.

**Figure 6.**
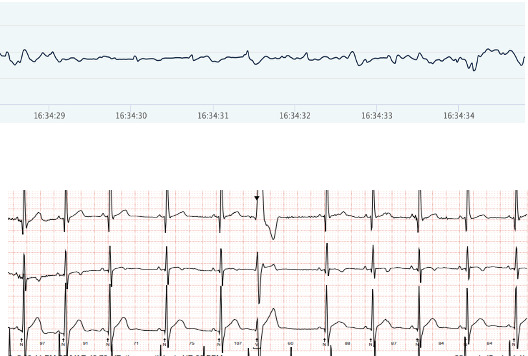
Example of electrocardiogram (ECG) rhythm with noise. Hexoskin recordings are depicted above and Holter ECG rhythms below.

### Questionnaire Results

**Figure 7.**
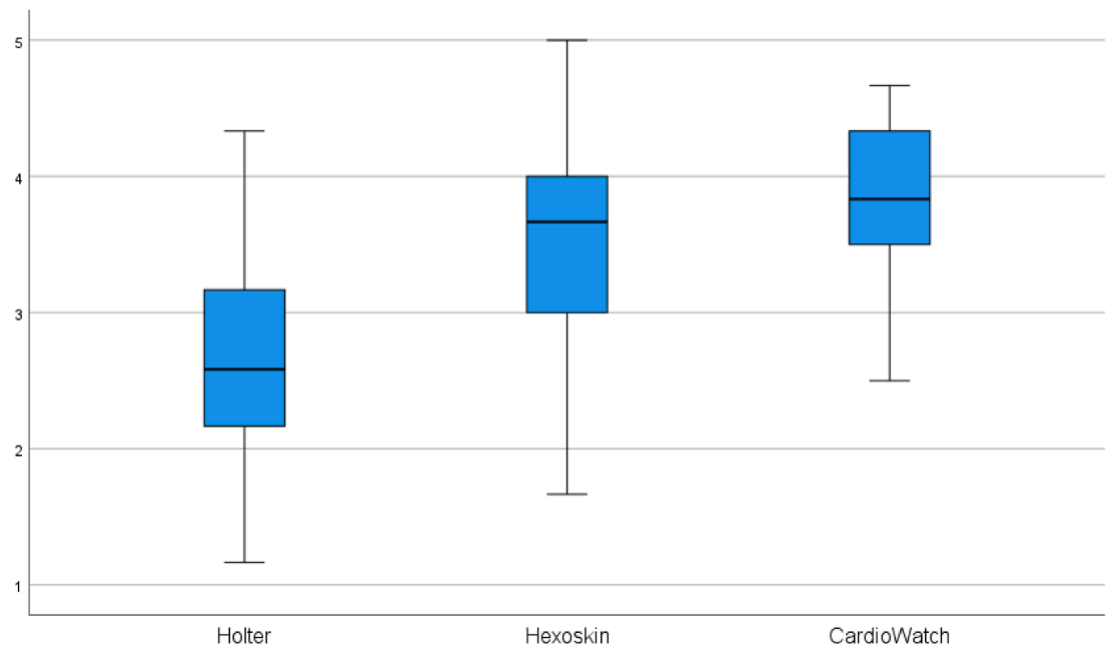
Boxplots questionnaire comfort scores.

The outcomes of the comfort questionnaire are shown in Table 2. Both wearables scored significantly higher on all questions compared to the Holter, reflecting better patient acceptance. The median scores were 3.8 (IQR 3.5‐4.3) for the CardioWatch and median 3.7 (IQR 3.0‐4.0) for the Hexoskin shirt, in contrast to median 2.5 (IQR 1.8‐3.2) for the Holter. This indicates that the CardioWatch and Hexoskin shirt were perceived as much more comfortable and pleasant than the Holter. Notably, all participants expressed a preference for wearing the CardioWatch and Hexoskin shirt instead of the Holter monitor. A participant removed the Hexoskin shirt prematurely during the study period. See Figure 7 for a visual representation of the comfort scores. An overview of all the question scores is provided in Multimedia Appendix 2.

### Accelerometry Analysis

Table 3 summarizes the mean accuracy of HR measurements across quantiles of movement intensity derived from participants’ accelerometry data, averaged over all participants. For both wearables, the accuracy of HR measurements decreased at higher quantiles, reflecting greater bodily movement intensity.

**Figure 8.**
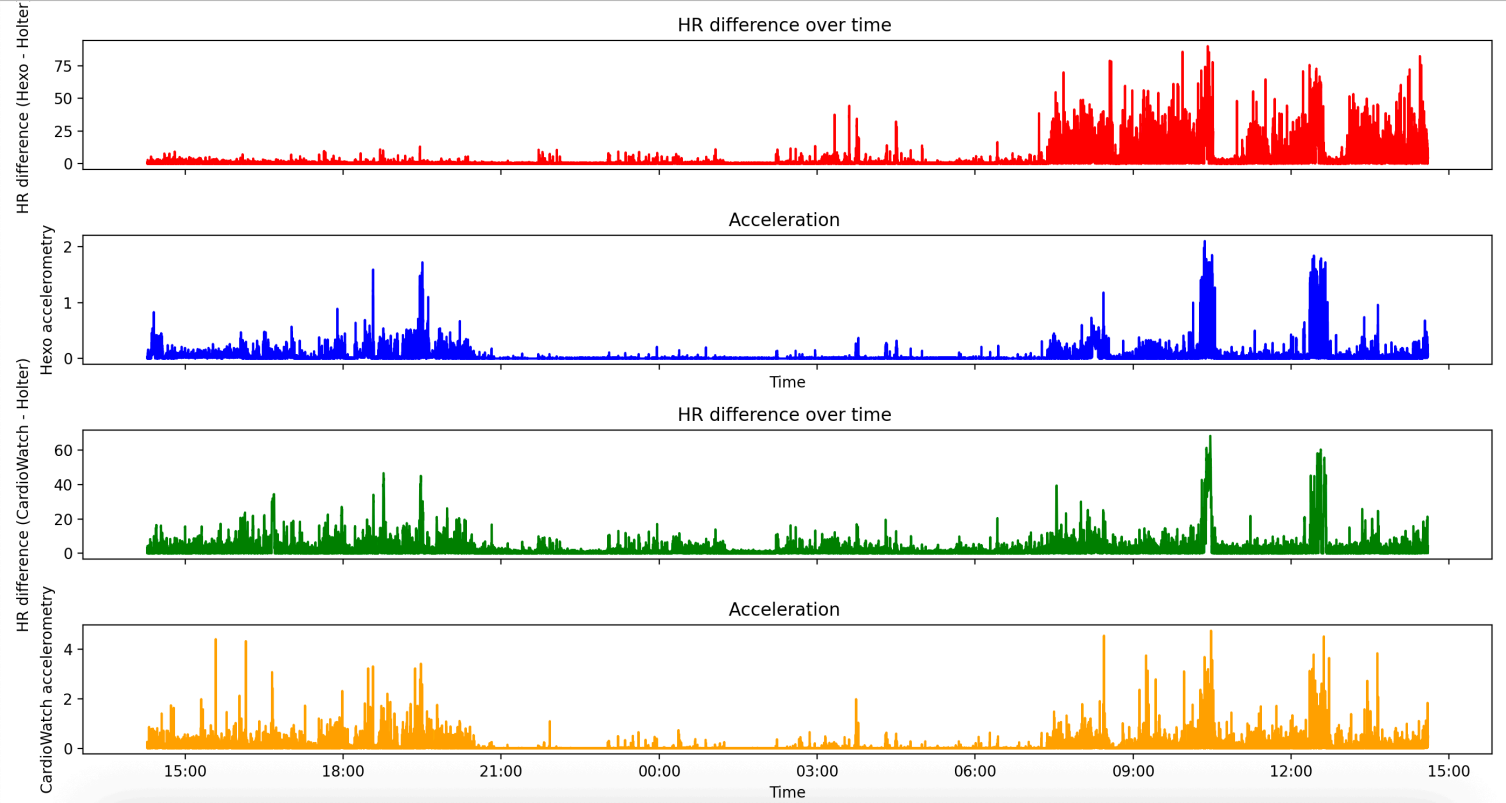
Differences in heart rate (HR) measurement versus accelerometry for Hexoskin (red and blue graph) and CardioWatch (green and yellow graph).

To further investigate potential age-related differences in activity and their impact on HR accuracy, a linear regression was performed with heart rate accuracy as the outcome, and age group and accelerometry as predictors, including an interaction term age group x accelerometry. There was no statistically significant interaction effect, which means that the relation between acceleration and accuracy did not differ between the two age groups. Figure 8 shows a visual representation of the effect of bodily movement on HR measurement accuracy for both wearables, where the difference in HR measurement between both the CardioWatch (depicted in green) and Hexoskin shirt (depicted in red) and Holter device are plotted in correspondence with the accelerometry data (orange and blue, respectively).

The individual accuracy of HR measurements in relation to the participants’ measures of accelerometry is shown in Multimedia Appendix 9.

**Table 3.** Accelerometry quantile-based accuracy measurement.

Quantile	CardioWatch (%), mean (SD)	Hexoskin (%), mean (SD)
First quantile	89.1 (8.5)	88.0 (10.7)
Second quantile	82.3 (9.3)	85.4 (11.1)
Third quantile	77.6 (11.4)	80.5 (13.8)
Fourth quantile	74.0 (16.9)	76.7 (17.5)

### Subgroup Analysis

Results of the subgroup analyses are shown in Table 4 and Multimedia Appendix 10. For the CardioWatch, the accuracy of HR measurements below the median heart rate was significantly higher than that of measurements above the median (90.9, SD 9.3% vs 79, SD 10.6%; *P*<.001). In addition, the accuracy of the CardioWatch was significantly higher during sleep than during wake time (90.1, SD 10.3% vs 82.1, SD 9.8%; *P*<.001).

Statistical analysis of HR measurements of the Hexoskin shirt showed a significantly greater accuracy in older children, in children with higher BMI, during higher HRs and during measurements at night. The accuracy scores per participant for the variables “Heart rate,” “Time of day,” and “Measuring period” are shown in MultimediaAppendices 11  12. The error outcomes for subgroup scores are shown in Table 5.

**Table 4.** Overview of subgroup analysis outcomes on accuracy for the CardioWatch and Hexoskin.

Accuracy	CardioWatch		Hexoskin	
	Mean (SD)	*P* value	Mean (SD)	*P* value
Sex		.71		.82
Female	85.4 (9.7)		86.8 (11.9)	
Male	84.3 (8.0)		87.9 (10.4)	
Age (years)		.76		.03
≤12	85.4 (7.4)		82.2 (11.5)	
>12	84.4 (9.6)		90.4 (9.7)	
BMI		.06		.002
≤19 kg/m2	81.3 (6.6)		82.0 (11.5)	
>19 kg/m2	87.3 (9.3)		92.3 (8.0)	
Diagnosis group		.88		.97
CHD^a^	83.7 (9.7)		86.8 (11.6)	
Arrhythmia	85.2 (8.4)		87.7 (10.6)	
Symptom	85.8 (7.6)		88.0 (12.2)	
Heart rate		<.001		<.001
≤Median heart rate	90.9 (9.3)		90.6 (14.0)	
>Median heart rate	79.0 (10.6)		84.5 (11.8)	
Time of day		<.001		<.001
Wake time	82.1 (9.8)		86.1 (10.9)	
Sleep time	90.1 (10.3)		90.8 (15.2)	
Wrist circumference		.92		—^b^
≤15.5 cm	84.9 (7.5)		—	
>15.5 cm	84.6 (10.7)		—	
Skin type (Fitzpatrick score)		.92		—
I and II	85.4 (7.8)		—	
III and IV	84.0 (8.3)		—	
V and VI	85.8 (7.6)		—	
Measuring period		.25		<.001
1st 12 hours	83.9 (10.7)		94.9 (7.4)	
2nd 12 hours	85.7 (8.4)		80.0 (16.7)	

a CHD: congenital heart disease.

b Not applicable.

**Table 5.** Error outcomes for the CardioWatch and Hexoskin.

Error	CardioWatch		Hexoskin	
	MAE^a^ (BPM^b^), mean (SD)	MAPE^c^ (%)	MAE (BPM), mean (SD)	MAPE (%)
Overall	4.9 (1.8)	5.5	4.3 (3.2)	4.9
HR^d^				
<Median HR	2.7 (1.4)	4.1	2.9 (3.2)	4.4
>Median HR	7.1 (2.8)	6.8	5.7 (4.1)	5.3
Time of day				
Wake time	6.0 (2.3)	6.2	5.0 (3.5)	5.1
Sleep time	2.9 (1.8)	4.1	2.7 (3.2)	4.1

a MAE: mean absolute error.

b BPM: beats per minute.

c MAPE: mean absolute percentage error.

d HR: heart rate.

## Discussion

### Principal Findings

This study assessed the accuracy and validity of HR measurements from the CardioWatch in 31 children and the accuracy and validity of both HR and rhythm measurements from the Hexoskin shirt in 36 children attending a pediatric cardiology outpatient clinic. Both the CardioWatch and Hexoskin shirt demonstrated high measurement accuracy and strong agreement with the Holter for HR assessment. The wearables showed minimal bias and reliable performance, suggesting they are valid tools for measuring HR in children with CHD or suspected arrhythmias. Nevertheless, Bland-Altman analyses indicated notable variability in measurement agreement, with accuracy decreasing at higher HRs. Consequently, both devices performed better during nighttime periods characterized by lower HRs compared to daytime periods, when activity levels and heart rates were higher. In addition, the accelerometry analysis demonstrated a clear inverse relationship between physical activity intensity and HR measurement accuracy. Taken together, these findings imply that the tested wearables may be particularly useful for continuous monitoring during rest or sleep rather than during vigorous physical activities, such as sports.

The Hexoskin shirt shows promising results for arrhythmia detection. However, noise and artifacts sometimes make it impossible to accurately classify rhythm recordings. This noise is caused by a combination of poor conduction due to the drying of conductive gel and shifting electrode placement. Future research should focus on these two factors to increase measurement accuracy. It is thereby important to realize that the Hexoskin shirt forms a 1-lead ECG signal, in contrast to the 3-lead ECG signal of the Holter device. The interpretation of the 1 lead signal will therefore always be inferior to that of the Holter device, as the latter usually provides at least one lead that allows for reliable rhythm interpretation.

Regarding the main study parameter, namely HR accuracy and validity, a previous study by Blok et al [26] reported higher accuracy (94.6%) and agreement (mean bias 0.06, LoA −3.89 to 3.77) for the CardioWatch compared to the findings in this study. They investigated the accuracy of the CardioWatch using a 12-lead ECG in older adults under resting conditions [26]. The difference in accuracy between the study by Blok et al [26] and ours can be explained by several factors, including their use of a signal qualifier and the absence of physical movement during their measurements. This lack of movement is known to influence PPG accuracy positively. Activity and motion artifacts can disturb the optical measurement of blood volume changes, leading to missing beats or inaccurate readings [8,25,27-32]. These factors likely explain the higher accuracy during nighttime and at lower heart rates in this study, as illustrated in MultimediaAppendices 11  12. The findings in this study are consistent with those of Hermans et al [28], who reported similar discrepancies in PPG accuracy in patients with atrial fibrillation. They attributed their higher observed accuracy during nighttime to factors such as reduced respiratory arrhythmia and fewer stimuli during the night [28]. To fully understand the effect of bodily movement on measurement accuracy in this study, the measures of accelerometry were analyzed in relation to HR accuracy. Unfortunately, not all CardioWatch data could be included in the analysis, as some data were missing. However, the outcomes confirmed that bodily movement negatively influences HR measurement accuracy. Multimedia Appendix 13 shows examples of 24-hour HR data with corresponding measures of accelerometry. As most wearables nowadays have built-in accelerometers, future research should include this accelerometry data to better understand the impact of bodily movements on measurement validity. In addition, improved algorithms that better filter out motion artifacts may help to enhance measurement accuracy [33].

The subgroup analysis revealed 2 notable findings. First, a positive but non-significant effect of BMI on CardioWatch performance was observed, contrasting with previous studies where higher BMI negatively impacted PPG accuracy [34,35]. Participants with a BMI >19 kg/m^2^ demonstrated higher accuracy, potentially due to a better fit from increased wrist fat tissue, similar to findings by Blok et al [26]. Likewise, the Hexoskin shirt showed higher HR accuracy in children with higher BMI, likely due to a tighter fit and subsequently less shifting of sensors, which could have improved ECG signal conduction. However, the BMI cutoff of 19 kg/m² may have oversimplified body composition differences, suggesting future research should use more detailed metrics to better understand their impact on accuracy. Second, Hexoskin accuracy declined over time, being nearly excellent during the first 12 hours— even showing resilience to bodily movements, as seen in Figure 8—but showed a significant reduction in the second 12-hour period. This decline likely resulted from the drying of conductive gel that was initially applied to the electrodes to improve signal conduction. As a result, noise increased, which was also visually observed in the signal during the second 12-hour measuring period. It is possible that the limited perspiration in children played a role too, as reduced moisture could have negatively affected signal transmission. To ensure consistent signal quality during long-term use, reapplying conductive gel every 12 hours is therefore recommended.

Besides HR monitoring, the Hexoskin shirt captures ECG rhythms. Of the 36 rhythm strips assessed, 31 were successfully classified by the blinded pediatric cardiologist, indicating that the Hexoskin can be useful for detecting minor arrhythmias, such as PVCs, PACs, and rhythms like ventricular trigeminy. However, further research is needed to evaluate the device’s accuracy in detecting more clinically relevant arrhythmias, such as SVT and bradycardias, as they did not occur during the measurements of this study. If validated, the Hexoskin could serve as a comfortable alternative to the Holter monitor for prolonged cardiac monitoring in children, offering a more practical and patient-friendly diagnostic tool for patients with infrequent appearing symptoms.

Although patients were instructed not to interfere with the smartphone, some of them closed and terminated the app. As a result, 4 measurements were discontinued and could not be used for analysis. This should be considered when using wearables for extended monitoring periods. Children are curious by nature and tend to explore how things work, which can lead to failed monitoring once they accidentally terminate a measurement. It may be good use to implement child locks or parental control on devices used for monitoring in children. Another contributor to the loss of CardioWatch data in this research was the shift to a different measurement frequency at night, from 1/s to 1/min, caused by a bug in the firmware. This shift occurred during the last 9 measurements. This shows that the firmware must be reliable, especially when monitoring a critical patient group, where missing data could have detrimental effects.

Inaccurate measurements can also occur due to poor wearable fit, resulting in a loss of skin contact. For optimal performance, especially during more intense activities, the CardioWatch must not be placed too loose to allow shifting or too tight to impede circulation [27]. The same holds for the Hexoskin shirt. Although the researchers visually checked the fitting of both devices at the start of the 24-hour measurement period, it is possible that the fit shifted during monitoring. Regarding wrist size, no differences in accuracy were observed between participants with smaller wrists (≤15.5 cm) and those with larger wrist circumferences (>15 cm). This may be attributed to the stretchable fabric used in the CardioWatch, which likely ensured a secure fit regardless of the wrist size. Manufacturers should consider using more adaptable materials to maintain consistent skin contact and reduce sensor errors.

Currently, there is no clear consensus on the methodology for determining heart rate measurement validity and accuracy. The MAPE is often adhered to as a measure of error which defines measurement validity and accuracy, but the threshold percentage of MAPE varies amongst studies. Some studies use a MAPE threshold of <5% to define validity and accuracy [36-38], where others use a MAPE threshold of <10% [21,29,39]. Besides MAPE, various studies measure validity and accuracy by calculating the percentage of measurements within 10% of the criterion measure values [21,40-43], which is in line with recommendations of the American National Standards Institute (ANSI) and Association for the Advancement of Medical Instrumentation guidelines (AAMI) [20]. Although the MAPE, ANSI, and AAMI methodologies may appear similar, they represent fundamentally different concepts and should not be conflated. To provide a comprehensive assessment of measurement validity and accuracy, this study reported both the MAPE and the accuracy percentage measured following the ANSI and AAMI guidelines. The lack of consensus on validity and accuracy guidelines complicates comparisons between studies, and methodological differences between wearable validation studies highlight the need for universal standards [16].

### Strengths and Limitations

This is one of the first studies that measured the validity of wearables in a cohort of children with CHD or (suspected) arrhythmias, while monitoring for prolonged duration under normal living conditions. Conducting this study outside the clinical setting enhanced its ecological validity by reflecting real-world conditions more accurately, which increased the generalizability of findings [26,44,45]. Another strength of this study is the included analysis of accelerometry, which shows the effect of bodily movement on measurement accuracy. On the other hand, a limitation of this study is the inability to fully validate the Hexoskin shirt’s diagnostic accuracy for measuring heart rhythm abnormalities, due to the small number of children with arrhythmias present in this study, and the fact that only minor abnormalities were observed. To determine its validity, it is essential that the Hexoskin shirt is also tested in detecting clinically relevant rhythm abnormalities, such as brady- and tachycardias, which were not observed during the measurements of this study. Nonetheless, the arrhythmias that were present were accurately recorded. In addition, the study’s cohort was relatively homogeneous, especially regarding BMI and skin type, which are known factors to influence accuracy. A more diverse study population would have made it possible to more accurately research the influence of these variables on measurement accuracy. Therefore, it is suggested that future studies adopt a multicenter study approach or include patients from different pediatric subgroups. Next to that, it is suggested to implement longer measuring periods to gain understanding of the technologies’ long-term performance and durability. Although this study included a relatively small sample size, the 24-hour measurement recorded over 100,000 repeated measurements, which ensured adequate statistical power. Future research should, in addition to addressing the aforementioned considerations, focus on enhancing measurement accuracy during increased bodily movements and minimizing missing data. With these valuable insights, new algorithms could be introduced that could improve measurement accuracy by reducing noise that is caused by physical movements.

### Conclusion

The CardioWatch and the Hexoskin shirt show good accuracy and strong correlation with the gold standard Holter ECG for monitoring heart rate in children attending the pediatric cardiology outpatient clinic. Both wearables offer improved comfort compared to the Holter, making them more suitable for prolonged use. The Hexoskin shirt’s ECG recordings show potential for effective ambulant rhythm monitoring. However, further research is needed to address the decline in accuracy during periods of bodily movement and higher heart rates, reduce missing data, and to validate the Hexoskin shirt’s capability to detect clinically significant arrhythmias in children. Conclusively, the wearables currently lack the validity required to replace clinical tools but could still contribute to valuable insights into heart rate and rhythm monitoring in noncritical situations, preferably during rest or sleep.

## Supplementary material

10.2196/70835 Multimedia Appendix 1 Questionnaire.

10.2196/70835 Multimedia Appendix 2 Patient satisfaction questionnaire outcomes.

10.2196/70835 Multimedia Appendix 3 Mean absolute error (MAE) and mean absolute percentage error (MAPE) equations.

10.2196/70835 Multimedia Appendix 4 Overview of percentage missing data for all participants.

10.2196/70835 Multimedia Appendix 5 Accuracy scores for the total 24-hour measurement period for all participants.

10.2196/70835 Multimedia Appendix 6 Error scores (mean absolute error and mean absolute percentage error) for the total 24-hour measurement period for all participants.

10.2196/70835 Multimedia Appendix 7 Concordance correlation coefficient scores for the total 24-hour measurement period for all participants.

10.2196/70835 Multimedia Appendix 8 Frequency of included arrhythmias.

10.2196/70835 Multimedia Appendix 9 Hexoskin heart rate measurement accuracy (%) per quantile of participants’ accelerometry data.

10.2196/70835 Multimedia Appendix 10 Concordance correlation coefficient outcomes for CardioWatch and Hexoskin.

10.2196/70835 Multimedia Appendix 11 Accuracy scores subgroup analysis for CardioWatch.

10.2196/70835 Multimedia Appendix 12 Accuracy scores subgroup analysis for Hexoskin.

10.2196/70835 Multimedia Appendix 13 Recordings of heart rate difference including measures of bodily movement.

## Abbreviations

AAMI: Association for the Advancement of Medical Instrumentations
ANSI: American National Standards Institute
BPM: beats per minute
CCC: Lin concordance correlation coefficient
CHD: congenital heart disease
ECG: electrocardiography
HR: heart rate
LoA: limits of agreements
MAE: mean absolute error
MAPE: mean absolute percentage error
PAC: premature atrial contraction
PPG: photoplethysmography
PVC: premature ventricular contraction
SVT: supraventricular tachycardia
VT: ventricular tachycardia
