# Supplementary material for: The Validation and Accuracy of Wearable Heart Rate Trackers in Children With Heart Disease: Prospective Cohort Study
Source: JMIR Form Res. 2025 Sep 30;9:e70835. doi: 10.2196/70835 (PMC12483337; doi:10.2196/70835)
Supplement: Multimedia Appendix 1 [file formative-v9-e70835-s001.docx]

Multimedia Appendix 1

Questionnaire

In Dutch, self developed

**
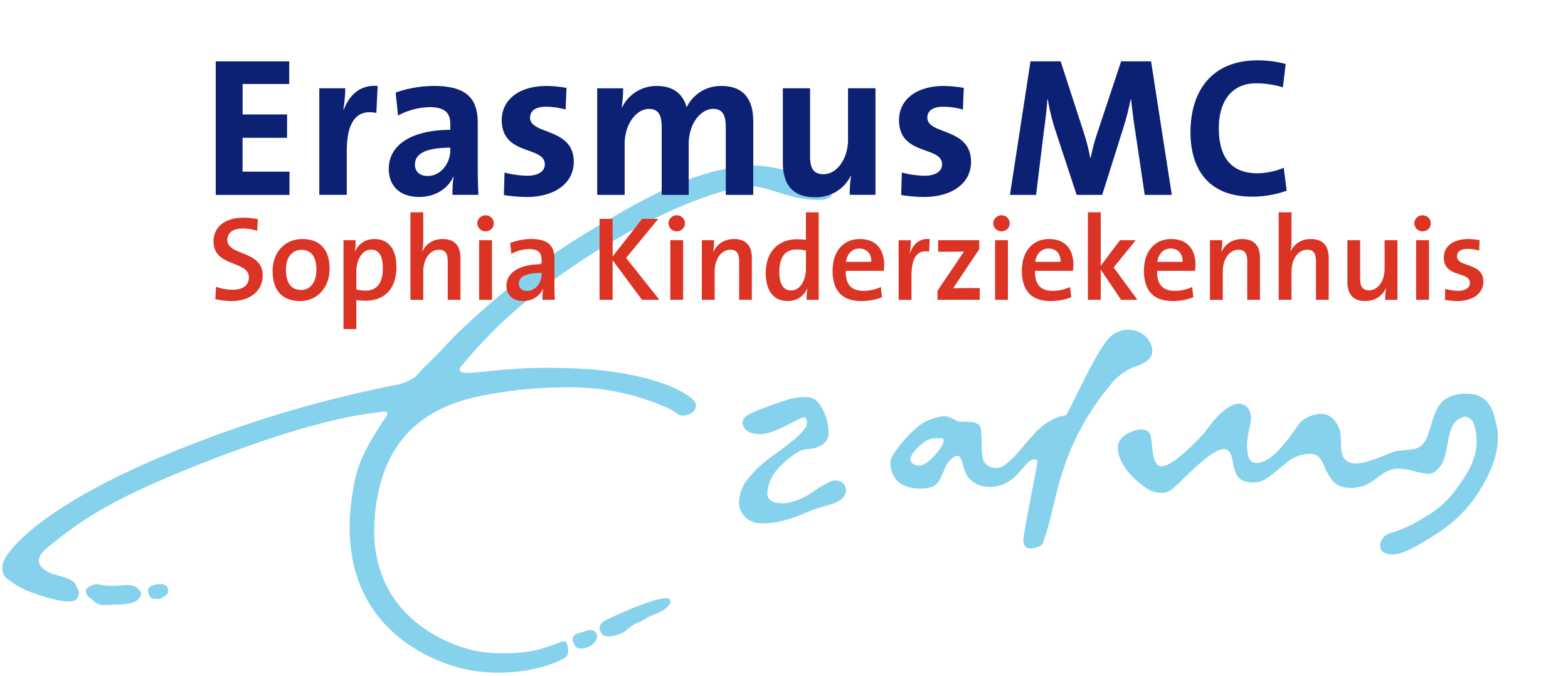
**

**Vragenlijst voor het onderzoek**

**Validatie van wearables.**

Augustus 2023

**Informatie over het invullen van de vragenlijst:**

**Waarom vul ik deze vragenlijst in?**

Omdat wij graag willen weten hoe jij het dragen van het polsbandje, het shirtje en de Holter vond.

**Wanneer moet ik hem invullen?**

Meteen nadat je de het polsbandje, het shirtje en de Holter af hebt gedaan.
Dit is een dag (24 uur) nadat we het polsbandje, het shirtje en de Holter bij je om hebben gedaan in het ziekenhuis.

**Hoe moet ik hem beantwoorden?**

Je krijgt 18 vragen waarin je uit vijf antwoorden kan kiezen. Kies altijd 1 antwoord per vraag. Heb je je antwoord gekozen, kleur dan het rondje in dat vóór jouw antwoord staat. Het is belangrijk dat je zo eerlijk mogelijk antwoord geeft. Het maakt niet uit of jouw antwoord negatief of positief is. Jouw mening is belangrijk voor ons.

Aan het eind kan je zelf kiezen of er nog iets is wat je graag aan ons wilt vertellen, dit mag jij (of je ouder) opschrijven in je eigen woorden. Daarna vragen wij je om te kiezen of je het polsbandje, het shirtje of de Holter liever draagt door er een 1, 2, of 3 voor te zetten. Een 1 draag je het liefst, en een 3 het minst graag.

**Wat als ik een antwoord niet weet?**

Probeer op alle vragen antwoord te geven. Vraag hulp aan je ouder of verzorger als je het zelf niet weet.

**Informatie voor ouders:**

Wanneer uw kind hulp nodig heeft bij het invullen van de vragenlijst, vragen wij u om de vragen samen met uw kind te beantwoorden. Vul daarbij altijd zo eerlijk mogelijk de ervaring van uw kind in.

**Eerst stellen we je wat vragen over het polsbandje:**

1.
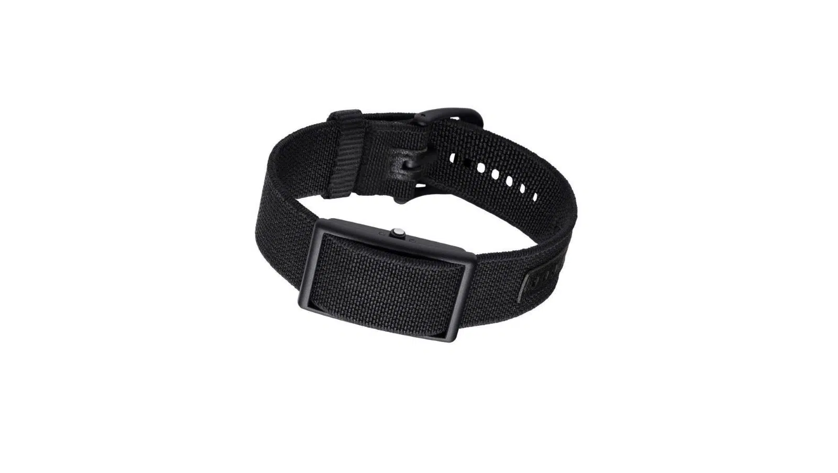
**Hoe vaak had je door dat je het polsbandje droeg?**

- Ik had het nooit door
- Ik had het bijna nooit door
- Ik had het soms door
- Ik had het vaak door
- Ik had het de hele tijd door

1. **Vond je het polsbandje prettig om te dragen?**

- Helemaal niet prettig
- Niet prettig
- Een beetje prettig
- Prettig
- Heel erg prettig

1. **Irriteerde het polsbandje je huid?**

- Helemaal geen irritatie
- Geen irritatie
- Een beetje irritatie
- Irritatie
- Heel veel irritatie

1. **Zat het polsbandje je in de weg bij bepaalde activiteiten? (Bijvoorbeeld: spelen, sporten, gamen, typen, afwassen).**

- Het zat me nooit in de weg
- Het zat me bijna nooit in de weg
- Het zat me soms in de weg
- Het zat me vaak in de weg
- Het zat me altijd in de weg

1. **Had je last van het polsbandje tijdens het slapen?**

- Ik had er helemaal geen last van
- Ik had er geen last van
- Ik had er een beetje last van
- Ik had er last van
- Ik had er heel veel last van

1. **Voel je je onzekerder of zelfverzekerder door het polsbandje?**

- Veel onzekerder



- Onzekerder



- Niet onzekerder, maar ook niet zelfverzekerder
- Zelfverzekerder
- Veel zelfverzekerder

**Nu stellen we je nog wat vragen over het shirtje:**


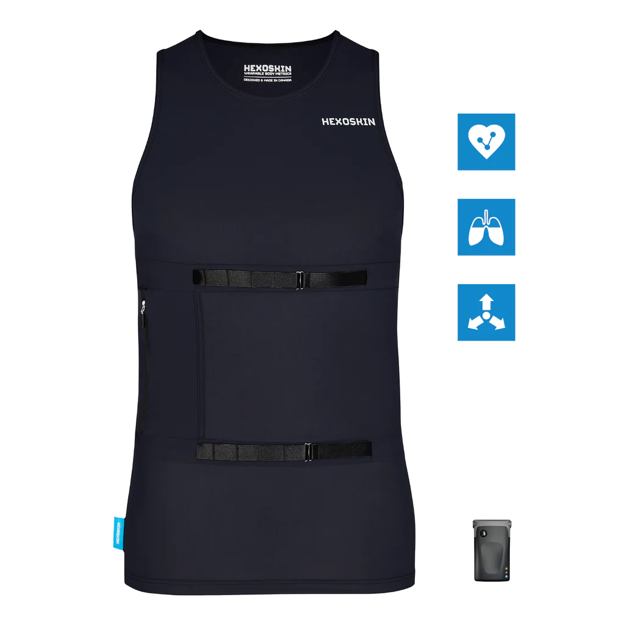


1. **Hoe vaak had je door dat je het shirtje droeg?**

- Ik had het nooit door
- Ik had het bijna nooit door
- Ik had het soms door
- Ik had het vaak door
- Ik had het de hele tijd door

1. **Vond je het shirtje prettig om te dragen?**

- Helemaal niet prettig
- Niet prettig
- Een beetje prettig
- Prettig
- Heel erg prettig

1. **Irriteerde het shirtje je huid?**

- Helemaal geen irritatie
- Geen irritatie
- Een beetje irritatie
- Irritatie
- Heel veel irritatie

1. **Zat het shirtje je in de weg bij bepaalde activiteiten? (Bijvoorbeeld: spelen, sporten, gamen, typen, afwassen)**

- Het zat me nooit in de weg
- Het zat me bijna nooit in de weg
- Het zat me soms in de weg
- Het zat me vaak in de weg
- Het zat me altijd in de weg

1. **Had je last van het shirtje tijdens het slapen?**

- Ik had er helemaal geen last van
- Ik had er geen last van
- Ik had er beetje last van
- Ik had er last van
- Ik had er heel veel last van



1. **Voel je je onzekerder of zelfverzekerder door het shirtje?**

- Veel onzekerder



- Onzekerder



- Niet onzekerder, maar ook niet zelfverzekerder
- Zelfverzekerder
- Veel zelfverzekerder

**Nu stellen we je ook nog wat vragen over de Holter (dit is het kastje met alle plakkers):**

1. **Hoe vaak had je door dat je de Holter droeg?**

-
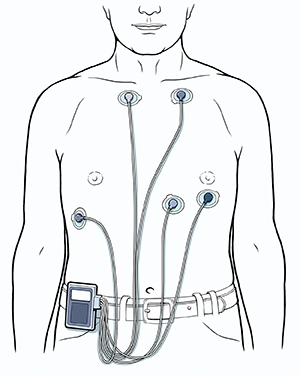
Ik had het nooit door
- Ik had het bijna nooit door
- Ik had het soms door
- Ik had het vaak door
- Ik had het de hele tijd door

1. **Vond je de Holter prettig om te dragen?**

- Helemaal niet prettig
- Niet prettig
- Een beetje prettig
- Prettig
- Heel erg prettig

1. **Irriteerde de Holter je huid?**

- Helemaal geen irritatie
- Geen irritatie
- Een beetje irritatie
- Irritatie
- Heel veel irritatie

1. **Zat de Holter je in de weg bij bepaalde activiteiten? (Bijvoorbeeld: spelen, sporten, gamen, typen, afwassen)**

- Het zat me nooit in de weg
- Het zat me bijna nooit in de weg
- Het zat me soms in de weg
- Het zat me vaak in de weg
- Het zat me altijd in de weg

1. **Had je last van de Holter tijdens het slapen?**

- Ik had er helemaal geen last van
- Ik had er geen last van
- Ik had er beetje last van
- Ik had er last van
- Ik had er heel veel last van



1. **Voel je je onzekerder of zelfverzekerder door de Holter?**

- Veel onzekerder



- Onzekerder



- Niet onzekerder, maar ook niet zelfverzekerder
- Zelfverzekerder
- Veel zelfverzekerder

**Je bent er bijna. Nog twee vragen:**

1. **Heb je problemen of juist fijne dingen ervaren? Die mag je in het groene vak opschrijven**
2. **Kan je de drie apparaatjes op plek 1 tot 3 zetten? Plek 1 is het apparaatje dat je het liefst zou gebruiken, plek 2 komt daarna en plek 3 voor het apparaatje dat je het minst graag gebruikt.**

- Het polsbandje
- Het shirtje
- De Holter

**Heel erg bedankt voor het meedoen aan ons onderzoek. We zijn superblij met je!**

**Het onderzoeksteam**
