# Supplementary material for: The Validation and Accuracy of Wearable Heart Rate Trackers in Children With Heart Disease: Prospective Cohort Study
Source: JMIR Form Res. 2025 Sep 30;9:e70835. doi: 10.2196/70835 (PMC12483337; doi:10.2196/70835)
Supplement: Multimedia Appendix 2 [file formative-v9-e70835-s002.docx]

Multimedia Appendix 2

Patient satisfaction questionnaire outcomes

IQR = Interquartile range H vs C = Holter vs CardioWatch, H vs H = Holter vs Hexoskin

| Question | Holter scores [median & IQR] | CardioWatch [median & IQR] | Z | p-value  [H vs C] | Hexoskin  [median & IQR] | Z | p-value  [H vs H] |  |
| --- | --- | --- | --- | --- | --- | --- | --- | --- |
| Overall: | 2.6 [2.1-3.2] | 3.8 [3.5-4.3] | -5,111 | <.001 | 3.7 [3.0-4.0] | -4,486 | <.001 | |
| Q1: How often did you notice wearing it? | 2.0 [1.0-2.0] | 3.0 [3.0-4.0] | -4,868 | <.001 | 3.0 [1.0-4.0] | -3,028 | .002 | |
| Q2: Was it pleasant to wear? | 2.0 [1.0-3.0] | 4.0 [3.0-4.0] | -4,972 | <.001 | 3.0 [2.0-4.0] | -4,169 | <.001 | |
| Q3: Did it irritate your skin? | 2.5 [2.0-4.0] | 4.0 [3.0-5.0] | -2,929 | .003 | 4.0 [3.75-5.0] | -4,366 | <.001 | |
| Q4: Did it bother during activities? | 3.0 [2.0-4.0] | 5.0[4.0-5.0] | -4,322 | <.001 | 5.0 [3.75-5.0] | -3,564 | <.001 | |
| Q5: Did it bother during sleep? | 3.0[2.0-4.0] | 5.0[3.75-5.0] | -4,228 | <.001 | 3.0 [3.0-5.0] | -2,333 | .020 | |
| Q6: Did you feel more secure or more insecure? | 3.0[2.75-3.0] | 3.0 [3.0-3.0] | -2,490 | .013 | 3.0[3.0-3.0] | -2,504 | .012 | |
| Final question: Which device do you prefer? |  |  |  | - |  |  |  | |
