# Supplementary material for: The Validation and Accuracy of Wearable Heart Rate Trackers in Children With Heart Disease: Prospective Cohort Study
Source: JMIR Form Res. 2025 Sep 30;9:e70835. doi: 10.2196/70835 (PMC12483337; doi:10.2196/70835)

Multimedia Appendix 3

MAE and MAPE equations

MAE is defined as the sum of errors, divided by the sample size:


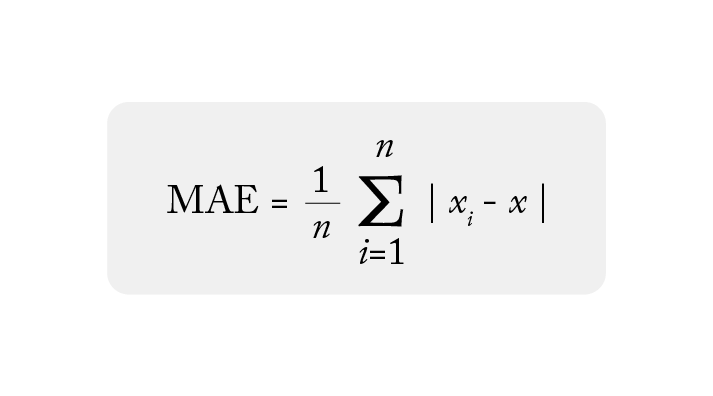


MAPE is defined as the (sum of) error(s), divided by (corresponding) criterion measure value, multiplied by 100


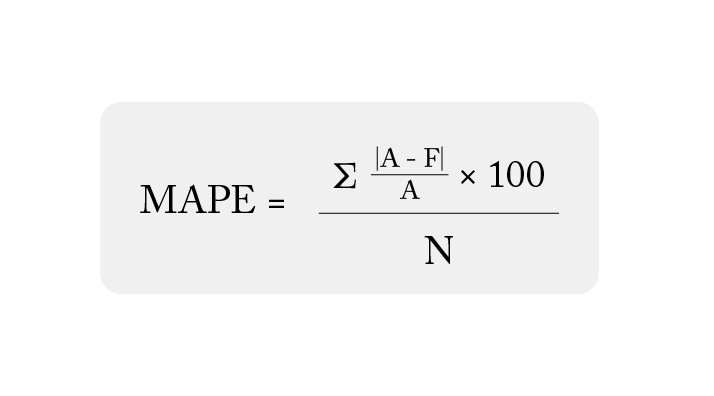

Supplement: Multimedia Appendix 3 [file formative-v9-e70835-s003.docx]
