# Supplementary material for: The Validation and Accuracy of Wearable Heart Rate Trackers in Children With Heart Disease: Prospective Cohort Study
Source: JMIR Form Res. 2025 Sep 30;9:e70835. doi: 10.2196/70835 (PMC12483337; doi:10.2196/70835)
Supplement: Multimedia Appendix 4 [file formative-v9-e70835-s004.docx]

Multimedia Appendix 4

Overview of percentage missing data for all participants

| Participant | Holter (%) | Corsano (%) |
| --- | --- | --- |
| 1 | 5,33 | 2,93 |
| 2 | 5,15 | 0,02 |
| 3 | 2,33 | 2,59 |
| 4 | 2,5 | 2,75 |
| 5 | 1,84 | - |
| 6 | 10,17 | 10,58 |
| 7 | 0,03 | 2,23 |
| 8 | 1,07 | 2,1 |
| 9 | 0,15 | 0,9 |
| 10 | 6,53 | 2,45 |
| 11 | 0,41 | 2,39 |
| 12 | 1,24 | 1,53 |
| 13 | - | - |
| 14 | 0,11 | 2,33 |
| 15 | 0,05 | 0,14 |
| 16 | 0,03 | 0,48 |
| 17 | 0,1 | 3,17 |
| 18 | - | - |
| 19 | 1,09 | 2,67 |
| 20 | 0,16 | 2,4 |
| 21 | 0,45 | 3,46 |
| 22 | 0,17 | 3,54 |
| 23 | 1,21 | - |
| 24 | 0,74 | 4,54 |
| 25 | 0,01 | 1,56 |
| 26 | 0,09 | - |
| 27 | 0,63 | 1,3 |
| 28 | 0,05 | - |
| 29 | - | - |
| 30 | 0,74 | - |
| 31 | 0,34 | - |
| 32 | 0,74 | - |
| 33 | 0,26 | - |
| 34 | 0,04 | - |
| 35 | 0,59 | - |
| 36 | 0,37 | - |
| 37 | 2,12 | - |
| 38 | 0,11 | - |
| 39 | 0,11 | - |

Due to a bug in the CardioWatch software, data of the last 9 participants showed a shift in sample rate at night from once per second to one measurement per minute. This resulted in missing timepoints and therefore a high percentage of missing data (36-40%). These participants were included in accuracy measurements but were left out in the calculation of missing data
