# Supplementary material for: The Validation and Accuracy of Wearable Heart Rate Trackers in Children With Heart Disease: Prospective Cohort Study
Source: JMIR Form Res. 2025 Sep 30;9:e70835. doi: 10.2196/70835 (PMC12483337; doi:10.2196/70835)
Supplement: Multimedia Appendix 5 [file formative-v9-e70835-s005.docx]

Multimedia Appendix 5

Accuracy scores for the total 24h measurement period for all participants.

|  | CardioWatch | Hexoskin |
| --- | --- | --- |
|  | Accuracy(%) | Accuracy(%) |
| Participant |  |  |
| 1 | 90,03 | 96,57 |
| 2 | 89,25 | 98,11 |
| 3 | 82,85 | 81,25 |
| 4 | 85,77 | 60,33 |
| 5 |  | 76,59 |
| 6 | 92,64 | 96,80 |
| 7 | 94,95 | 98,67 |
| 8 | 90,14 | 98,36 |
| 9 | 90,19 | 75,98 |
| 10 | 74,90 | 95,70 |
| 11 | 91,83 | 84,51 |
| 12 | 95,78 | 84,68 |
| 13 |  |  |
| 14 | 84,95 | 93,63 |
| 15 | 90,24 | 97,54 |
| 16 | 97,39 | 99,44 |
| 17 | 67,05 | 69,36 |
| 18 |  |  |
| 19 | 83,15 | 86,50 |
| 20 | 93,14 | 95,30 |
| 21 | 75,36 | 66,19 |
| 22 | 80,97 | 70,20 |
| 23 |  | 92,24 |
| 24 | 85,64 | 78,85 |
| 25 | 98,67 | 98,95 |
| 26 |  | 94,11 |
| 27 | 83,93 | 85,74 |
| 28 |  | 98,29 |
| 29 |  |  |
| 30 |  | 97,78 |
| 31 | 73,80 | 67,75 |
| 32 | 81,83 | 91,78 |
| 33 | 70,33 | 92,84 |
| 34 | 95,19 | 94,21 |
| 35 | 77,59 | 81,96 |
| 36 | 71,04 | 84,61 |
| 37 | 73,47 | 82,11 |
| 38 | 80,04 | 84,20 |
| 39 | 86,35 | 96,58 |
