# Supplementary material for: The Validation and Accuracy of Wearable Heart Rate Trackers in Children With Heart Disease: Prospective Cohort Study
Source: JMIR Form Res. 2025 Sep 30;9:e70835. doi: 10.2196/70835 (PMC12483337; doi:10.2196/70835)
Supplement: Multimedia Appendix 6 [file formative-v9-e70835-s006.docx]

Multimedia Appendix 6

Error scores (MAE & MAPE) for the total 24h measurement period for all participants.

|  | CardioWatch | CardioWatch | Hexoskin | Hexoskin |
| --- | --- | --- | --- | --- |
|  | MAE(BPM) | MAPE(%) | MAE(BPM | MAPE(%) |
| Participant |  |  |  |  |
| 1 | 4.04 | 4.39 | 1,58 | 1,79 |
| 2 | 3.79 | 4.06 | 1,11 | 1,31 |
| 3 | 5.16 | 5.56 | 6,39 | 6,84 |
| 4 | 5.52 | 5.13 | 13,53 | 18,04 |
| 5 |  |  | 8,19 | 8,68 |
| 6 | 4.15 | 5.03 | 1,36 | 1,71 |
| 7 | 2.83 | 2.83 | 1,29 | 1,33 |
| 8 | 4.10 | 3.80 | 1,27 | 1,32 |
| 9 | 4.02 | 4.22 | 8,21 | 8,82 |
| 10 | 7.21 | 7.61 | 2,13 | 2,21 |
| 11 | 3.63 | 3.77 | 4,30 | 4,82 |
| 12 | 2.93 | 2.75 | 7,03 | 6,22 |
| 13 |  |  |  |  |
| 14 | 4.28 | 5.01 | 2,32 | 2,53 |
| 15 | 2.92 | 3.88 | 1,29 | 1,77 |
| 16 | 1.84 | 2.35 | 0,75 | 1,01 |
| 17 | 6.64 | 9.14 | 5,34 | 7,67 |
| 18 |  |  |  |  |
| 19 | 6.30 | 6.68 | 4,18 | 6,75 |
| 20 | 3.35 | 3.69 | 2,34 | 2,79 |
| 21 | 6.95 | 9.17 | 9,15 | 12,65 |
| 22 | 6.21 | 6.57 | 7,91 | 8,53 |
| 23 |  |  | 3,11 | 3,17 |
| 24 | 5.77 | 5.39 | 8,65 | 9,43 |
| 25 | 1.59 | 1.92 | 0,89 | 1,05 |
| 26 |  |  | 2,63 | 2,47 |
| 27 | 6.04 | 5.26 | 6,13 | 4,97 |
| 28 |  |  | 1,07 | 1,43 |
| 29 |  |  |  |  |
| 30 |  |  | 1,65 | 2,07 |
| 31 | 4.81 | 7.62 | 6,16 | 10,02 |
| 32 | 6.73 | 6.00 | 3,76 | 3,45 |
| 33 | 5.85 | 8.64 | 2,33 | 3,34 |
| 34 | 2.40 | 2.61 | 2,11 | 1,95 |
| 35 | 7.02 | 7.20 | 7,22 | 6,43 |
| 36 | 6.91 | 9.81 | 4,41 | 6,08 |
| 37 | 8.40 | 7.65 | 8,19 | 6,47 |
| 38 | 6.65 | 6.41 | 5,95 | 5,56 |
| 39 | 4.38 | 4.96 | 1,57 | 1,77 |
