# Supplementary material for: The Validation and Accuracy of Wearable Heart Rate Trackers in Children With Heart Disease: Prospective Cohort Study
Source: JMIR Form Res. 2025 Sep 30;9:e70835. doi: 10.2196/70835 (PMC12483337; doi:10.2196/70835)
Supplement: Multimedia Appendix 7 [file formative-v9-e70835-s007.docx]

Multimedia Appendix 7

Concordance correlation coefficient scores for the total 24h measurement period for all participants.

CCC = Concordance correlation coefficient CI = confidence interval

|  | CardioWatch |  |  | Hexoskin |  |  |
| --- | --- | --- | --- | --- | --- | --- |
|  | CCC | Lower  95% CI | Upper 95% CI | CCC | Lower  95% CI | Upper 95% CI |
| Participant |  |  |  |  |  |  |
| 1 | 0,959 | 0,958 | 0,960 | 0,990 | 0,990 | 0,990 |
| 2 | 0,943 | 0,941 | 0,944 | 0,992 | 0,992 | 0,993 |
| 3 | 0,886 | 0,884 | 0,887 | 0,723 | 0,718 | 0,728 |
| 4 | 0,946 | 0,945 | 0,947 | 0,767 | 0,764 | 0,771 |
| 5 |  |  |  | 0,723 | 0,717 | 0,728 |
| 6 | 0,847 | 0,840 | 0,853 | 0,992 | 0,992 | 0,992 |
| 7 | 0,947 | 0,945 | 0,948 | 0,985 | 0,984 | 0,985 |
| 8 | 0,901 | 0,899 | 0,904 | 0,988 | 0,987 | 0,989 |
| 9 | 0,946 | 0,945 | 0,948 | 0,723 | 0,719 | 0,727 |
| 10 | 0,949 | 0,948 | 0,950 | 0,988 | 0,987 | 0,989 |
| 11 | 0,910 | 0,907 | 0,912 | 0,857 | 0,854 | 0,860 |
| 12 | 0,941 | 0,940 | 0,942 | 0,461 | 0,454 | 0,468 |
| 13 |  |  |  |  |  |  |
| 14 | 0,727 | 0,722 | 0,732 | 0,809 | 0,804 | 0,815 |
| 15 | 0,952 | 0,951 | 0,953 | 0,986 | 0,985 | 0,987 |
| 16 | 0,953 | 0,950 | 0,955 | 0,994 | 0,994 | 0,994 |
| 17 | 0,940 | 0,939 | 0,941 | 0,956 | 0,955 | 0,957 |
| 18 | 0,792 | 0,786 | 0,797 | 0,902 | 0,897 | 0,906 |
| 19 |  |  |  |  |  |  |
| 20 | 0,971 | 0,970 | 0,971 | 0,966 | 0,964 | 0,967 |
| 21 | 0,482 | 0,475 | 0,489 | 0,426 | 0,419 | 0,432 |
| 22 | 0,902 | 0,900 | 0,904 | 0,861 | 0,859 | 0,863 |
| 23 |  |  |  | 0,906 | 0,903 | 0,909 |
| 24 | 0,771 | 0,767 | 0,775 | 0,548 | 0,541 | 0,555 |
| 25 | 0,985 | 0,984 | 0,985 | 0,988 | 0,987 | 0,989 |
| 26 |  |  |  | 0,900 | 0,897 | 0,903 |
| 27 | 0,759 | 0,755 | 0,763 | 0,600 | 0,594 | 0,606 |
| 28 |  |  |  | 0,962 | 0,958 | 0,966 |
| 29 |  |  |  |  |  |  |
| 30 |  |  |  | 0,94 | 0,936 | 0,944 |
| 31 | 0,927 | 0,926 | 0,929 | 0,831 | 0,827 | 0,836 |
| 32 | 0,850 | 0,847 | 0,852 | 0,914 | 0,911 | 0,916 |
| 33 | 0,775 | 0,771 | 0,779 | 0,914 | 0,911 | 0,917 |
| 34 | 0,949 | 0,947 | 0,950 | 0,859 | 0,854 | 0,863 |
| 35 | 0,874 | 0,871 | 0,877 | 0,757 | 0,753 | 0,761 |
| 36 | 0,612 | 0,606 | 0,618 | 0,729 | 0,723 | 0,734 |
| 37 | 0,887 | 0,886 | 0,889 | 0,779 | 0,775 | 0,783 |
| 38 | 0,811 | 0,809 | 0,814 | 0,740 | 0,736 | 0,745 |
| 39 | 0,929 | 0,928 | 0,931 | 0,974 | 0,973 | 0,975 |
