# Supplementary material for: The Validation and Accuracy of Wearable Heart Rate Trackers in Children With Heart Disease: Prospective Cohort Study
Source: JMIR Form Res. 2025 Sep 30;9:e70835. doi: 10.2196/70835 (PMC12483337; doi:10.2196/70835)
Supplement: Multimedia Appendix 8 [file formative-v9-e70835-s008.docx]

Multimedia Appendix 8

Frequency of included arrythmias

| Abnormality | # appearances |
| --- | --- |
|  |  |
| 1st degree AV block |  |
|  | 3 |
| Premature VE |  |
|  | 7 |
| Sinus rhythm |  |
|  | 11 |
| Trigiminy |  |
|  | 1 |
| SVE |  |
|  | 9 |
| Ventricular Bigeminy |  |
|  | 1 |
| Ventricular Triplet |  |
|  | 1 |
| Arrhythmia |  |
|  | 3 |
