# Supplementary material for: The Validation and Accuracy of Wearable Heart Rate Trackers in Children With Heart Disease: Prospective Cohort Study
Source: JMIR Form Res. 2025 Sep 30;9:e70835. doi: 10.2196/70835 (PMC12483337; doi:10.2196/70835)
Supplement: Multimedia Appendix 9 [file formative-v9-e70835-s009.docx]

Multimedia Appendix 9

Hexoskin HR measurement accuracy (%) per quantile of participants’ accelerometry data.

|  | **0** | **1** | **2** | **3** |
| --- | --- | --- | --- | --- |
|  |  |  |  |  |
| **P001** | 96,9 | 95,6 | 93,3 | 91,9 |
| **P002** | 98,7 | 98,5 | 96,8 | 94,1 |
| **P003** | 78,7 | 73,1 | 67,6 | 53,9 |
| **P004** | 62 | 66,3 | 70,3 | 67,1 |
| **P005** | 77,9 | 72,6 | 65,1 | 55,9 |
| **P006** | 98,6 | 99,4 | 99,2 | 99,7 |
| **P007** | 98,5 | 98 | 97,1 | 95,3 |
| **P008** | 97 | 96,2 | 94,5 | 94,3 |
| **P009** | 76,8 | 68,3 | 65,8 | 50,7 |
| **P010** | 97,3 | 97 | 95,7 | 88,5 |
| **P011** | 78,7 | 80,6 | 78,6 | 70,1 |
| **P012** | 86,1 | 80,9 | 74,9 | 60,2 |
| **P013** | 95,4 | 83,3 | 67,1 |  |
| **P014** | 98,4 | 96,6 | 94,1 | 91 |
| **P015** | 99,2 | 99,2 | 99,4 |  |
| **P016** | 74,3 | 79,2 | 79,2 | 89,4 |
| **P017** | 87,1 | 88,9 | 91,8 | 96 |
| **P018** | 97,4 | 96,6 | 92 | 93,7 |
| **P019** | 69 | 72,8 | 66,5 |  |
| **P020** | 63,7 | 62,4 | 62,6 | 69,5 |
| **P021** | 91,9 | 89,8 | 82,4 | 83,6 |
| **P022** | 75,2 | 74,8 | 72,9 | 74,1 |
| **P023** | 98,6 | 98,1 | 96,2 | 96,4 |
| **P024** | 97,6 | 95,6 | 92,1 | 73,1 |
| **P025** | 91,3 | 83,9 | 59,7 | 36,8 |
| **P026** | 97,4 | 96,4 | 97,9 |  |
| **P027** | 98,7 | 98,8 | 98,5 | 94,6 |
| **P028** | 82,3 | 82,9 | 75,3 | 72,1 |
| **P029** | 90 | 83,2 | 78,2 | 81,8 |
| **P030** | 91,3 | 88,7 | 80,8 |  |
| **P031** | 87,7 | 80,6 | 71,4 | 79,6 |
| **P032** | 83,2 | 76,1 | 61,1 | 57,1 |
| **P033** | 83,6 | 73,8 | 65,3 | 53,4 |
| **P034** | 90 | 78,9 | 67,2 | 53,1 |
| **P035** | 82,1 | 72,5 | 58,2 |  |
| **P036** | 97,1 | 94,2 | 88 | 83,3 |

CardioWatch HR measurement accuracy (%) per quantile of participants accelerometry data. A high number of patient data was missing, including the last 9 participants that were measured with the bug in the firmwaren.

| **Proefpersoon** | **0** | **1** | **2** | **3** |
| --- | --- | --- | --- | --- |
|  |  |  |  |  |
| **P001** |  |  |  |  |
| **P002** | 91,2 | 83,5 | 80,7 |  |
| **P003** | 84,4 | 80,1 | 77,4 |  |
| **P004** |  |  |  |  |
| **P005** |  |  |  |  |
| **P006** |  |  |  |  |
| **P007** | 95,2 | 91,9 | 90 |  |
| **P008** | 93,3 | 67,2 |  |  |
| **P009** | 93,1 | 85,5 | 77,3 |  |
| **P010** | 78,8 | 71,4 | 68,1 | 70,9 |
| **P011** | 94,8 | 88,9 | 78,4 |  |
| **P012** | 96,6 | 95,6 | 93,1 |  |
| **P013** | 96,4 | 82,5 |  |  |
| **P014** | 90,9 | 82,8 | 80 |  |
| **P015** | 97,5 | 97,7 | 96,8 | 92,2 |
| **P016** | 64,2 | 66,1 | 72,2 |  |
| **P017** | 83,2 | 74,5 | 66,9 | 58,8 |
| **P018** | 94,4 | 89,9 | 86,5 |  |
| **P019** | 78,8 | 80,8 | 73,4 |  |
| **P020** | 83 | 76,7 | 70,9 |  |
| **P021** |  |  |  |  |
| **P022** | 89 | 80,2 | 64,3 |  |
| **P023** | 99,2 | 97,5 | 96,1 |  |
| **P024** |  |  |  |  |
| **P025** | 91,6 | 77,5 | 57,3 |  |
| **P026** |  |  |  |  |
| **P027** | 85,8 | 74,9 | 67,3 |  |
| **P028** |  |  |  |  |
| **P029** |  |  |  |  |
| **P030** |  |  |  |  |
| **P031** |  |  |  |  |
| **P032** |  |  |  |  |
| **P033** |  |  |  |  |
| **P034** |  |  |  |  |
| **P035** |  |  |  |  |
| **P036** |  |  |  |  |
