# Supplementary material for: The Validation and Accuracy of Wearable Heart Rate Trackers in Children With Heart Disease: Prospective Cohort Study
Source: JMIR Form Res. 2025 Sep 30;9:e70835. doi: 10.2196/70835 (PMC12483337; doi:10.2196/70835)
Supplement: Multimedia Appendix 10 [file formative-v9-e70835-s010.docx]

Multimedia Appendix 10

Concordance correlation coefficient outcomes for the CardioWatch and Hexoskin.

| **CCC** | CardioWatch |  | Hexoskin |  |
| --- | --- | --- | --- | --- |
|  | Correlation | 95% Lower- Upper CI | Correlation | 95% Lower-Upper CI |
|  |  |  |  |  |
| Heart rate |  |  |  |  |
| <median HR  >median HR | 0.773  0.740 | 0.767-0.780  0.735-0.745 | 0.700  0.712 | 0.688-0.705  0.714-0.725 |
| Time of day |  |  |  |  |
| waketime  sleep time | 0.815  0.794 | 0.811-0.819  0.787-0.801 | 0.788  0.812 | 0.783-0.792  0.804-0.819 |
