# Supplementary material for: The Validation and Accuracy of Wearable Heart Rate Trackers in Children With Heart Disease: Prospective Cohort Study
Source: JMIR Form Res. 2025 Sep 30;9:e70835. doi: 10.2196/70835 (PMC12483337; doi:10.2196/70835)
Supplement: Multimedia Appendix 11 [file formative-v9-e70835-s011.docx]

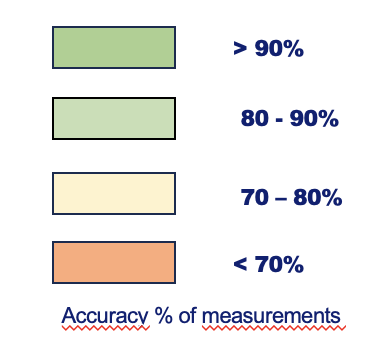
Multimedia Appendix 11

Accuracy scores subgroup analysis CardioWatch

|  | Heart rate |  | Time of day |  | Measuring | period |
| --- | --- | --- | --- | --- | --- | --- |
|  | <median | >median | Waketime | Sleeptime | 1^st^ 12h | 2^nd^ 12h |
| Participant |  |  |  |  |  |  |
| 1 | 95,68 | 84,47 | 87,74 | 90,19 | 87,02 | 93,04 |
| 2 | 95,69 | 82,86 | 85,42 | 96,85 | 84,73 | 93,77 |
| 3 | 91,68 | 74,20 | 78,37 | 91,47 | 84,24 | 81,46 |
| 4 | 95,45 | 76,11 | 79,96 | 94,73 | 80,02 | 91,52 |
| 5 |  |  |  |  |  |  |
| 6 | 91,31 | 93,92 | 92,22 | 93,66 | 96,13 | 89,15 |
| 7 | 98,05 | 91,85 | 92,56 | 99,04 | 94,63 | 95,27 |
| 8 | 99,50 | 81,21 | 82,11 | 98,72 | 85,14 | 95,15 |
| 9 | 96,10 | 84,61 | 87,20 | 97,97 | 91,70 | 88,68 |
| 10 | 81,20 | 68,80 | 71,94 | 83,01 | 74,61 | 75,19 |
| 11 | 98,47 | 85,45 | 88,29 | 98,18 | 93,38 | 90,29 |
| 12 | 98,88 | 92,88 | 96,40 | 95,42 | 97,87 | 93,69 |
| 13 |  |  |  |  |  |  |
| 14 | 89,90 | 80,40 | 78,62 | 97,32 | 76,79 | 93,12 |
| 15 | 97,05 | 83,60 | 86,65 | 95,97 | 90,99 | 89,49 |
| 16 | 99,48 | 95,36 | 97,03 | 96,72 | 98,13 | 96,65 |
| 17 | 63,46 | 70,63 | 64,90 | 74,77 | 60,32 | 73,77 |
| 18 | 95,35 | 71,83 | 80,23 | 90,67 | 77,29 | 89,02 |
| 19 |  |  |  |  |  |  |
| 20 | 95,67 | 90,67 | 93,16 | 92,48 | 94,66 | 91,63 |
| 21 | 85,95 | 66,27 | 81,25 | 60,56 | 78,54 | 72,19 |
| 22 | 87,78 | 74,16 | 77,59 | 88,11 | 84,84 | 77,11 |
| 23 |  |  |  |  |  |  |
| 24 | 95,13 | 76,60 | 78,23 | 94,37 | 83,66 | 87,63 |
| 25 | 99,88 | 97,52 | 98,33 | 99,48 | 99,17 | 98,17 |
| 26 |  |  |  |  |  |  |
| 27 | 97,62 | 71,10 | 74,72 | 97,27 | 91,85 | 76,00 |
| 28 |  |  |  |  |  |  |
| 29 |  |  |  |  |  |  |
| 30 |  |  |  |  |  |  |
| 31 | 65,00 | 82,35 | 83,64 | 55,00 | 73,18 | 74,43 |
| 32 | 94,42 | 69,24 | 77,55 | 89,20 | 83,57 | 80,09 |
| 33 | 83,89 | 57,72 | 62,91 | 91,40 | 58,14 | 82,51 |
| 34 | 97,79 | 92,67 | 94,87 | 96,37 | 96,76 | 93,61 |
| 35 | 80,91 | 74,31 | 75,05 | 89,56 | 77,56 | 77,62 |
| 36 | 76,87 | 65,51 | 64,39 | 85,75 | 73,89 | 68,20 |
| 37 | 87,46 | 59,90 | 67,14 | 88,59 | 69,22 | 77,72 |
| 38 | 88,65 | 71,83 | 78,09 | 85,26 | 75,68 | 84,41 |
| 39 | 92,68 | 80,02 | 87,25 | 84,16 | 87,32 | 85,38 |
| **Mean** | 90,87 | 78,97 | 82,06 | 90,07 | 83,90 | 85,68 |
| **SD** | 9,34 | 10,57 | 9,76 | 10,33 | 10,72 | 8,41 |
