# Supplementary material for: The Validation and Accuracy of Wearable Heart Rate Trackers in Children With Heart Disease: Prospective Cohort Study
Source: JMIR Form Res. 2025 Sep 30;9:e70835. doi: 10.2196/70835 (PMC12483337; doi:10.2196/70835)
Supplement: Multimedia Appendix 12 [file formative-v9-e70835-s012.docx]

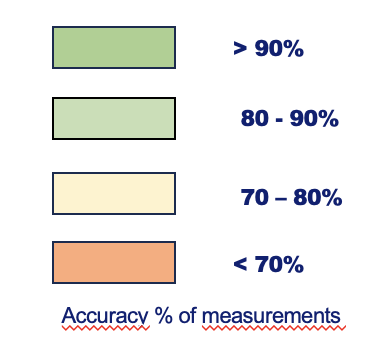
Multimedia Appendix 12

Accuracy scores subgroup analysis Hexoskin

|  | Heart rate |  | Time of day |  | Measuring | period |
| --- | --- | --- | --- | --- | --- | --- |
|  | <median | >median | Waketime | Sleeptime | 1^st^ 12h | 2^nd^ 12h |
| Participant |  |  |  |  |  |  |
| 1 | 98,67 | 94,51 | 95,88 | 96,82 | 95,52 | 97,63 |
| 2 | 99,06 | 97,17 | 97,66 | 99,04 | 97,52 | 98,69 |
| 3 | 90,72 | 71,97 | 74,98 | 92,81 | 90,22 | 72,29 |
| 4 | 48,42 | 72,11 | 69,49 | 47,75 | 72,38 | 48,28 |
| 5 | 82,39 | 71,18 | 74,92 | 86,51 | 80,36 | 72,82 |
| 6 | 94,14 | 99,42 | 96,88 | 97,35 | 99,02 | 94,59 |
| 7 | 99,55 | 97,79 | 98,20 | 99,58 | 99,18 | 98,16 |
| 8 | 99,96 | 96,82 | 96,98 | 99,80 | 98,08 | 98,63 |
| 9 | 86,05 | 66,48 | 73,89 | 84,79 | 95,97 | 55,98 |
| 10 | 97,07 | 94,38 | 95,03 | 97,03 | 97,69 | 93,71 |
| 11 | 87,38 | 81,76 | 76,75 | 98,69 | 99,87 | 69,16 |
| 12 | 96,08 | 74,01 | 82,35 | 93,98 | 97,96 | 71,40 |
| 13 |  |  |  |  |  |  |
| 14 | 99,42 | 88,29 | 92,57 | 96,38 | 93,96 | 93,30 |
| 15 | 98,98 | 96,13 | 97,24 | 97,98 | 98,67 | 96,40 |
| 16 | 99,85 | 99,04 | 99,69 | 99,03 | 99,60 | 99,28 |
| 17 | 54,14 | 84,54 | 76,09 | 54,85 | 72,76 | 65,96 |
| 18 | 86,79 | 86,23 | 85,85 | 87,49 | 97,45 | 75,55 |
| 19 |  |  |  |  |  |  |
| 20 | 94,86 | 95,73 | 96,42 | 96,23 | 98,46 | 92,14 |
| 21 | 71,02 | 62,03 | 63,72 | 65,66 | 86,36 | 46,01 |
| 22 | 78,40 | 61,98 | 65,23 | 79,46 | 91,94 | 48,45 |
| 23 | 98,33 | 86,51 | 91,60 | 94,16 | 98,48 | 92,14 |
| 24 | 80,55 | 77,25 | 78,61 | 83,53 | 94,76 | 62,95 |
| 25 | 99,91 | 98,04 | 98,61 | 99,82 | 99,85 | 98,06 |
| 26 | 99,03 | 89,51 | 92,35 | 99,53 | 96,78 | 91,44 |
| 27 | 98,99 | 73,67 | 74,56 | 99,60 | 99,65 | 71,84 |
| 28 | 98,26 | 98,31 | 99,43 | 97,37 | 99,76 | 96,82 |
| 29 |  |  |  |  |  |  |
| 30 | 97,7 | 97,85 | 98,62 | 99,19 | 99,35 | 96,21 |
| 31 | 50,92 | 84,11 | 80,98 | 39,28 | 79,60 | 55,89 |
| 32 | 98,14 | 85,42 | 88,09 | 98,83 | 99,25 | 84,31 |
| 33 | 97,21 | 88,77 | 91,57 | 96,87 | 96,51 | 89,17 |
| 34 | 99,64 | 88,93 | 90,60 | 99,67 | 100,00 | 88,42 |
| 35 | 94,49 | 69,56 | 78,69 | 96,15 | 97,25 | 66,66 |
| 36 | 92,58 | 77,04 | 77,39 | 97,59 | 98,44 | 70,78 |
| 37 | 98,12 | 66,60 | 76,18 | 98,25 | 94,65 | 69,56 |
| 38 | 94,39 | 74,51 | 77,29 | 97,73 | 98,96 | 69,44 |
| 39 | 99,56 | 93,61 | 94,64 | 99,35 | 99,70 | 93,47 |
| **Mean** | 89,84 | 83,80 | 85,23 | 90,04 | 94,88 | 78,59 |
| **SD** | 14,71 | 12,00 | 10,98 | 16,16 | 7,39 | 17,27 |
