# Supplementary material for: The Validation and Accuracy of Wearable Heart Rate Trackers in Children With Heart Disease: Prospective Cohort Study
Source: JMIR Form Res. 2025 Sep 30;9:e70835. doi: 10.2196/70835 (PMC12483337; doi:10.2196/70835)

Multimedia Appendix 13

Recordings of heart rate difference including measures of bodily movement

Patiënt 11


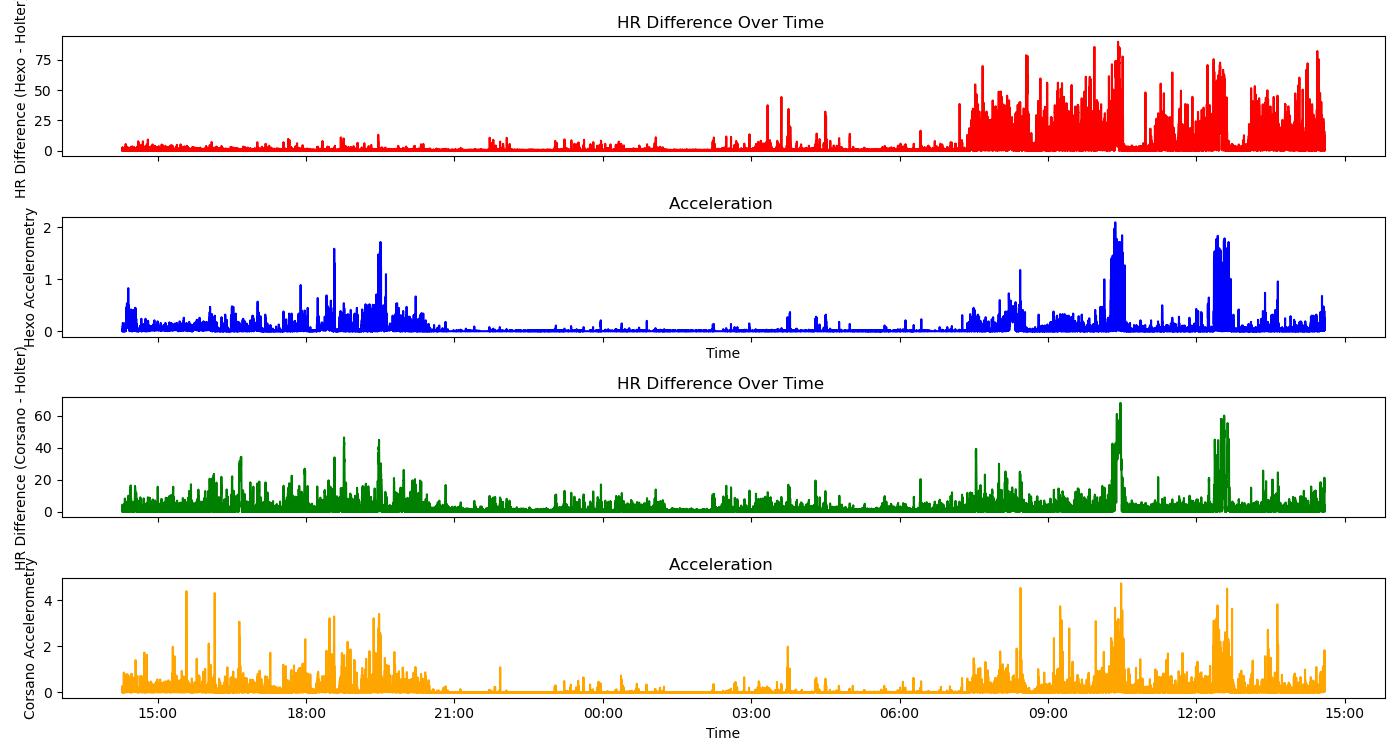


Patiënt 12


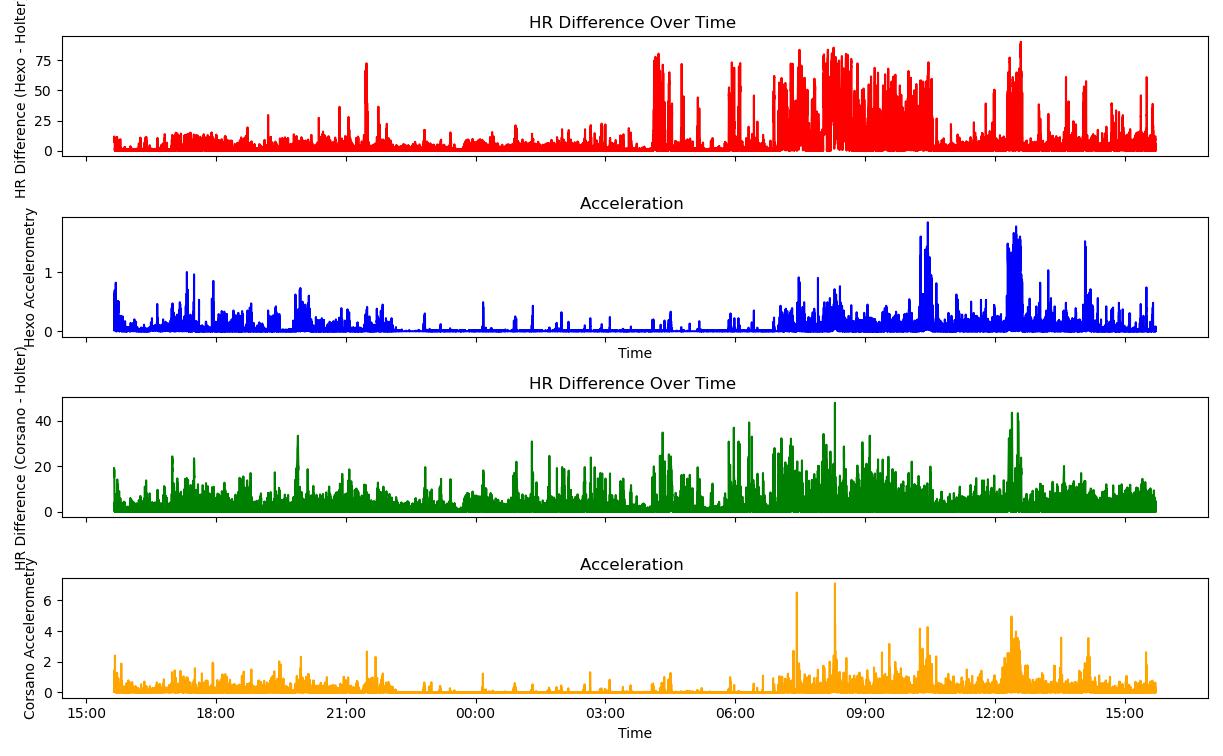


Patiënt 15


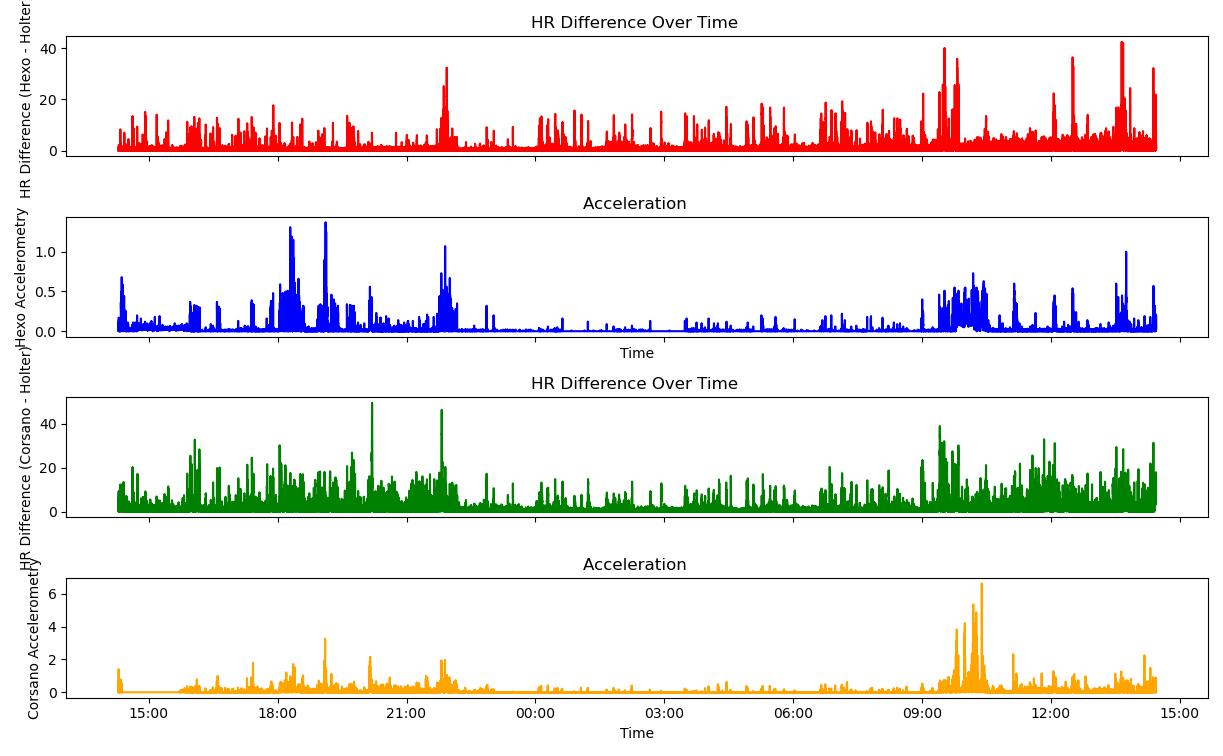


Patiënt 25


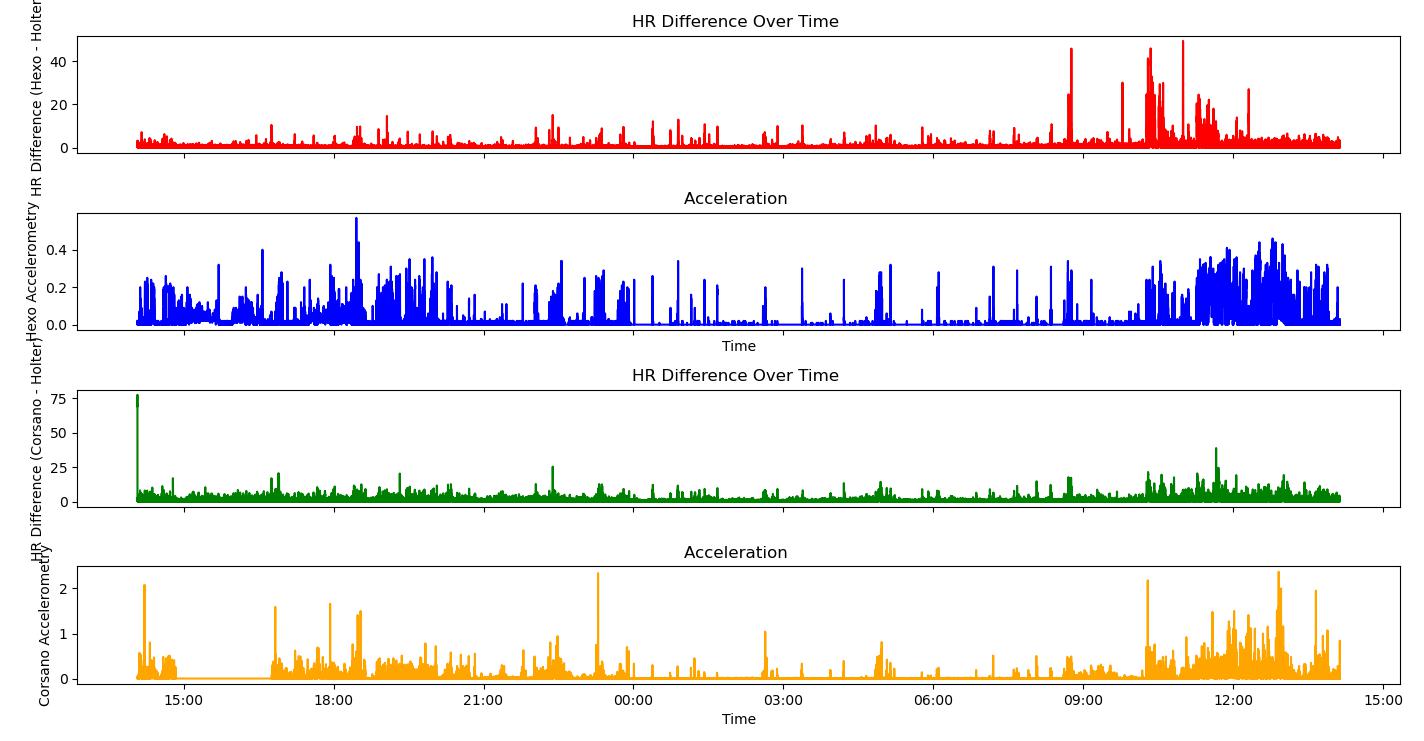

Supplement: Multimedia Appendix 13 [file formative-v9-e70835-s013.docx]
